# Effects of Exercise Snacks on Cardiometabolic Health and Body Composition in Adults: A Systematic Review and Meta‐Analysis

**DOI:** 10.1111/sms.70114

**Published:** 2025-08-14

**Authors:** Ke‐wen Wan, Zi‐han Dai, Po‐san Wong, Wendy Y. Huang, Evander Fung‐chau Lei, Jonathan P. Little, Feng‐Chang Lin, Bjorn T. Tam

**Affiliations:** ^1^ Faculty of Arts and Social Sciences, Academy of Wellness and Human Development Hong Kong Baptist University Kowloon Tong, Hong Kong China; ^2^ Dr. Stephen Hui Research Centre for Physical Recreation and Wellness Hong Kong Baptist University Kowloon Tong, Hong Kong China; ^3^ Department of Sports Science and Physical Education The Chinese University of Hong Kong Hong Kong China; ^4^ School of Health and Exercise Sciences University of British Columbia Kelowna British Columbia Canada; ^5^ Department of Biostatistics University of North Carolina at Chapel Hill Chapel Hill North Carolina USA

**Keywords:** cardiometabolic health, exercise snacks, intermittent exercise, meta‐analysis

## Abstract

This systematic review and meta‐analysis aims to investigate the impact of exercise snacks (ExSn), which involve incorporating short bursts of high‐intensity physical activity into daily routines, on improving cardiometabolic health and body composition in adults. Six online databases [PubMed, Web of Science, Embase, Cochrane Central Register of Controlled Trials (CENTRAL), CINAHL, and Scopus] were searched from inception through 22 May 2025, and relevant randomized controlled trials (RCTs) and non‐randomized controlled trials (non‐RCTs) were identified. Outcomes were analyzed using standardized mean differences and mean differences with 95% confidence intervals. Subgroup analyses were conducted based on physical activity levels and duration for each bout of ExSn. The GRADE scale was used to assess evidence certainty, while the revised Cochrane risk‐of‐bias tool (RoB 2) was used to evaluate the quality of RCTs, and the Risk of Bias In Non‐randomized Studies of Interventions (ROBINS‐I) tool was used for non‐RCTs. Twelve RCTs and two non‐RCTs, involving a total of 483 adults, were selected for the systematic review; 13 studies were included in the meta‐analysis. Among the RCTs, 2 studies showed a high risk of bias, and 10 showed some concerns. For the non‐RCTs, 1 study had a moderate risk of bias, and 1 had a serious risk of bias. The ExSn group showed significant improvements in maximal oxygen uptake (SMD = 1.43, 95% CI 0.61 to 2.25, *p* < 0.001) and peak power output (SMD = 0.68, 95% CI 0.00 to 1.36, *p* = 0.050), and reductions in total cholesterol (SMD = −0.65, 95% CI −1.18 to −0.11, *p* = 0.018) and LDL cholesterol (SMD = −0.65, 95% CI −1.22 to −0.09, *p* = 0.023). No significant differences were found for body weight, body fat, HDL cholesterol, or triglycerides. ExSn significantly enhances cardiometabolic health, especially in physically inactive adults. As a novel, time‐efficient approach, ExSn can be easily integrated into daily routines, offering a practical solution for sedentary and inactive individuals or those with limited time. These findings highlight its potential as a widely applicable public health strategy, warranting further research on long‐term effects and broader applications.

**PROSPERO Registration Number:** CRD42024554446

**Abbreviations**
%BF: body fat percentage
BW: body weight
CI: confidence interval
ExSn: exercise snacks
HDL‐C: high‐density lipoprotein cholesterol
LDL‐C: low‐density lipoprotein cholesterol
MD: mean difference
PICOS: population, intervention, comparison, outcomes, and study design
PPO: peak power output
PRISMA: Preferred Reporting Items for Systematic Reviews and Meta‐Analysis
RCT: randomized controlled trial
ROB 2: revised Cochrane risk of bias tool
SD: standard deviation
SMD: standardized mean difference
TC: total cholesterol
TG: triglycerides
VO_2_max: maximal oxygen uptake

## 1 Introduction

Physical inactivity is a significant global public health concern, with approximately one in four adults worldwide failing to meet the recommended levels of physical activity (PA) [1]. Previous research has shown that physical inactivity is associated with an increased risk of developing chronic health conditions such as cardiovascular diseases, diabetes, obesity, and certain types of cancer [2, 3, 4]. Being physically inactive can negatively impact mental health by increasing the risk of developing feelings of stress, anxiety, and depression [5, 6]. Furthermore, research suggests that middle‐aged and older adults who lead physically inactive lifestyles encounter various barriers, including physical limitations and environmental constraints, as well as psychological factors such as perceptions of effort and energy [7, 8]. Therefore, designing effective and accessible PA interventions and programs to address these barriers is critical to promoting PA levels and overall health in adults.

Exercise snacks (ExSn), defined as short and efficient bursts of exercise spread across the day, are emerging as a convenient and effective strategy to increase PA levels, reduce sedentary behavior, and promote overall health and well‐being [9, 10, 11, 12, 13]. Most studies commonly employ exercise sessions lasting 1–2 min, although some studies have extended the duration up to 10 min, depending on individual factors such as age and tolerance levels [9, 14]. Importantly, the intensity of ExSn is relative to the individual's fitness level, age, and maximal heart rate, allowing activities to be tailored to meet the needs of diverse populations, including older adults or those with reduced physical capacity [15, 16]. Normally, ExSn encompasses planned and structured activities, including aerobic exercises, strength‐based exercises, balance‐focused activities, and combinations thereof, as well as intermittent activity integrated into daily routines, such as fast walking during commuting, all aimed at providing short bursts of exercise within everyday activities. By requiring minimal time and effort, ExSn can help mitigate barriers such as physical limitations and perceptions of excessive effort, while their flexibility allows older adults to perform them in various environments, reducing environmental constraints. Similarly, the concept of vigorous‐intensity lifestyle physical activity (VILPA) also emphasizes brief, high‐intensity activity, but it differs in that VILPA occurs spontaneously during daily tasks, such as climbing stairs or carrying heavy groceries, rather than through structured and intentional efforts [17, 18]. While both ExSn and VILPA share the benefit of delivering high‐intensity activity in short bouts, ExSn is distinguished by its planned and systematic nature, which allows for better tracking, adherence, and the formation of consistent physical activity habits over time. This structured approach makes ExSn particularly promising for individuals seeking practical and sustainable ways to integrate PA into their daily routines.

Emerging research, including a scoping review and individual studies, supports the safety, feasibility, and benefits of ExSn across various populations, including the elderly, healthy adults, and individuals with conditions such as obesity and diabetes [14, 19, 20]. Engaging in brief bursts of intense physical activity, even in small amounts throughout the day, has been linked to a reduced risk of mortality [21]. Furthermore, previous meta‐analyses have demonstrated that both continuous and accumulated patterns of exercise yield similar benefits in terms of fitness, blood pressure, and metabolic markers [22, 23]. However, short‐bout exercises have been found to enhance exercise adherence when compared to long‐bout [24, 25, 26]. Therefore, compared to continuous exercise routines that may require more time commitment, ExSn offers a more convenient and feasible solution for office workers who spend long hours sitting in the office and for individuals who prefer shorter, more manageable bursts of physical activity.

In recent years, there has been a growing number of randomized controlled trials (RCTs) investigating various types of ExSn [9, 16]. Despite this increasing interest, no systematic review with meta‐analysis has been conducted to evaluate the effectiveness of ExSn on cardiometabolic health and body composition. Furthermore, there is a lack of research examining the effects of ExSn on adults with different PA levels, including both physically active individuals and those who are physically inactive. Understanding how exercise snacks may influence metabolic health and body composition in these distinct populations is crucial for tailoring exercise recommendations and interventions to meet the specific needs and circumstances of individuals with varying activity levels. Therefore, the primary objective of this systematic review and meta‐analysis is to comprehensively investigate the potential effects of ExSn on cardiometabolic health and body composition, with a particular focus on targeted studies conducted among physically active and inactive adults.

## 2 Methods

### 2.1 Registration

This systematic review and meta‐analysis was registered with PROSPERO (CRD42024554446) and conducted in accordance with the PRISMA guidelines and the Cochrane Handbook for Systematic Reviews of Interventions on 15 June 2024 [27].

### 2.2 Search Strategy

A comprehensive literature search was conducted from inception through 22 May 2025, across six electronic databases: PubMed, Web of Science, Embase, Cochrane Central Register of Controlled Trials (CENTRAL), CINAHL, and Scopus. The search strategy was developed using a combination of keywords and MeSH terms, with details provided in the Table S5. The search was restricted to full‐text articles published in English and involving human subjects. Additional studies were identified by screening the reference lists of articles obtained through our systematic search.

### 2.3 Eligibility Criteria and Study Selection

**FIGURE 1:**
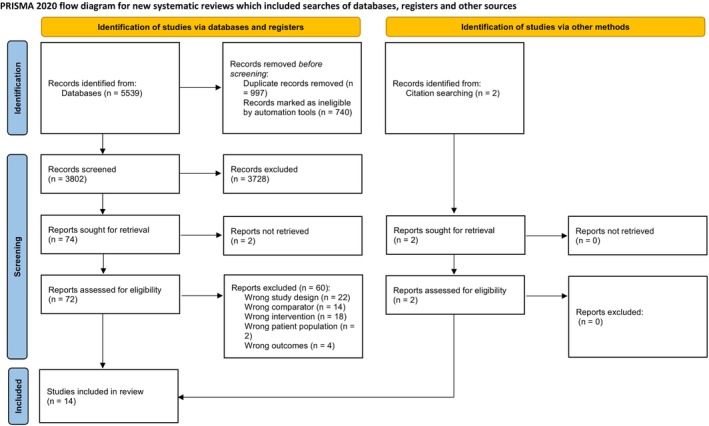
Flowchart of publications included in systematic review and meta‐analysis (PRISMA diagram). PRISMA, Preferred Reporting Items for Systematic Reviews and Meta‐Analyses.

Figure 1 presents the study selection process, illustrating the steps taken to identify and include relevant studies in the analysis. EndNote 21 (Clarivate Analytics) was used to import and manage all search results, with duplicates subsequently removed. Two review authors (KW and ZD) conducted an initial screening of the titles and abstracts of the identified studies to identify those that potentially met the inclusion criteria. Subsequently, the full texts of these potentially relevant articles were independently reviewed by the same two authors to determine their eligibility for inclusion in the review. In cases where discrepancies or disagreements arose between the two authors, a third author (BT) was consulted to facilitate resolution and reach a consensus. The selection of eligible studies followed the Population, Intervention, Comparison, Outcomes, and Study (PICOS) criteria [28], as outlined below: (i) Population: a population of apparently healthy individuals aged ≥ 18 years, (ii) Interventions: groups that performed ExSn (brief, efficient bursts of exercise throughout the day, typically lasting 1–2 min but occasionally up to 10 min, aimed at boosting physical activity levels and overall well‐being), (iii) Comparator: groups that continued with their usual lifestyle, (iv) Outcomes: outcome measures included cardiometabolic health outcomes [maximal oxygen uptake (VO_2_max), peak power output (PPO), total cholesterol (TC), high‐density lipoprotein cholesterol (HDL‐C), low‐density lipoprotein cholesterol (LDL‐C), triglycerides (TG)], and body composition [body weight (BW), body fat percentage (%BF)], (v) Study design: Studies adopted either a RCT or non‐randomized controlled trial (non‐RCT) design.

#### 2.3.1 Data Extraction

The data extraction procedures adhered to the guidelines outlined in the Cochrane Collaboration Handbook [29]. Two authors (KW and PW) independently extracted data from the included studies, entering the information into an electronic spreadsheet (Excel, 2023). The extracted data encompassed the following study characteristics: study details (first author, publication year, country), participant characteristics, study design, study duration, description of interventions, duration of “exercise snacks” per session, main outcomes, and key findings. The characteristics of the included studies are summarized in Table S1. Forest plots were generated by conducting meta‐analyses using the change means and standard deviations (SDs) or mean differences and their corresponding SDs for each outcome. In cases where change values were not reported, we reached out to the corresponding authors of the articles to obtain the necessary data. When obtaining complete data was not feasible, we employed a correlation coefficient (*R*) to calculate the standard deviation of the pre‐post change (ΔSD). This correlation coefficient was derived based on the calculated coefficients. The ΔSD was calculated using the following formula, as previously recommended [29]:

$$\mathrm{\Delta SD}=\sqrt{\left({\mathrm{SD}}^{2}\text{baseline}+{\mathrm{SD}}^{2}\text{final}\right)-\left(2\times R\times \text{SDbaseline}\times \text{SDfinal}\right)}$$

To ensure the accuracy of the extracted data, a third author (ZD) conducted a thorough review and verification of the data. To assess the reliability of the study selection process, inter‐rater agreement was evaluated using Cohen's Kappa statistic. This statistical measure accounts for agreement occurring by chance, providing a robust assessment of inter‐rater reliability.

### 2.4 Quality Assessment

The assessment of the risk of bias in each included study was conducted by two reviewers (KW and ZD). For randomized controlled trials (RCTs), the revised Cochrane risk‐of‐bias tool for randomized trials (RoB 2) was used, and studies were rated as “low risk,” “some concerns,” or “high risk” of bias [30]. For non‐randomized controlled trials (non‐RCTs), the Risk Of Bias In Non‐randomized Studies of Interventions (ROBINS‐I) tool was applied, with studies rated as “low risk,” “moderate risk,” “serious risk,” or “critical risk” of bias [31]. To ensure that the risk of bias assessment is outcome‐specific, we grouped outcomes into three categories based on their characteristics and potential sources of bias, as described in Tables S6 and S7. Both reviewers independently assessed each study, and in cases of discrepancies, a third researcher (BT) was consulted to reach a consensus.

### 2.5 Certainty of Evidence

The assessment of the certainty of evidence for each outcome was conducted by two authors (KW and ZD) using the Grading of Recommendations Assessment, Development, and Evaluation (GRADE) protocol [32]. The GRADE approach involves assigning ratings that span from very low to high levels of certainty, enabling the evaluation of the quality and strength of the evidence supporting each outcome. Various criteria, including risk of bias, consistency, directness, precision, and publication bias, were considered to determine the potential downgrading of certainty and strength of recommendations [33]. The authors engaged in discussions to ensure consensus on the interpretation of the findings during the GRADE assessment.

### 2.6 Synthesis Methods and Statistical Analysis

Statistical analyses were conducted using R (version 4.3.3) with the metafor package 7.0–0 [34, 35]. One author (KW) performed the data analysis and synthesis, and meta‐analysis was performed when data were available from at least two reports. Cardiometabolic health‐related outcomes were analyzed using standardized mean differences (SMDs) with 95% confidence intervals (CIs), whereas body composition parameters were analyzed using mean differences (MDs) with 95% CIs [36]. Random‐effect models were employed for the calculations, taking into account the potential heterogeneity in clinical or methodological factors that could have influenced the outcomes. Effect sizes were computed to evaluate and compare the effects of ExSn versus the control group for the respective outcomes. Statistical heterogeneity was assessed using *I* ^2^ values, which represent the proportion of total variation across studies that is due to heterogeneity rather than chance. The *I* ^2^ values were categorized as low (0%–25%), moderate (26%–50%), substantial (51%–75%), and high (more than 75%) [29]. These measures provided insights into the degree of heterogeneity present among the included studies. To enhance the robustness of the findings, a series of sensitivity analyses were conducted to assess the influence of each individual study. These sensitivity analyses were performed using a leave‐one‐out approach, systematically excluding one study at a time and examining the impact on the results. Funnel plots were used to visually assess publication bias when at least ten studies were included in the meta‐analysis, and Egger's linear regression test was employed to investigate funnel plot asymmetry. Subgroup analysis was performed as follows: (i) participants' PA level (physically active and physically inactive), (ii) duration for each bout of ExSn (less or equal to 2 min and more than 2 min). Meta‐regression analyses were conducted to examine the influence of potential moderators (age, duration of each ExSn bout, and duration of the trial) on the outcomes (peak power output and maximal oxygen uptake). The regression coefficients (*β*) and corresponding *p*‐values were used to assess the significance of these relationships.

### 2.7 Equity, Diversity, and Inclusion Statement

Our study included a diverse group of healthy adults from various genders, socioeconomic backgrounds, and cultures, using randomized controlled trials globally.

Our author team comprises student and senior researchers, including a Master's student, PhD candidate, assistant, associate, and full professors. Team members originate from Hong Kong, Canada, and the USA, representing multiple disciplines. We considered accessibility, regional differences, and socioeconomic levels in our inclusive data collection methods, emphasizing diverse perspectives despite no authors being from marginalized backgrounds.

## 3 Results

### 3.1 Study Selection

A total of 5539 records were identified for screening from six electronic databases. After removing duplicates, 3802 records remained. Of these, 3728 were excluded based on title and abstract screening for not meeting the inclusion criteria. Seventy‐two full‐text articles were reviewed in depth, and 60 were excluded for the following reasons: (i) wrong study design (*n* = 22), (ii) inappropriate control group (*n* = 14), (iii) did not use exercise snacks as intervention (*n* = 18), (iv) subjects did not meet inclusion criteria (*n* = 2), and (v) did not have the aimed outcomes (*n* = 4). Two additional papers were included through citation searching. Ultimately, 14 papers were selected for the systematic review, and 13 were included in the meta‐analysis. Detailed results of the literature search are presented in the PRISMA flow diagram (Figure 1). Two independent reviewers (KW and ZD) screened 74 studies for inclusion in the meta‐analysis. Inter‐rater reliability was assessed using Cohen's Kappa statistic, which yielded a value of 0.83 (*κ* = (P_o–P_e)/(1−P_e) = (0.9459–0.6730)/(1−0.6730)), indicating almost perfect agreement between the reviewers. Any discrepancies were resolved through discussion, resulting in the final inclusion of 14 studies.

### 3.2 Characteristics of Included Studies

Table 1 provides an overview of the characteristics of the 13 included studies [10, 37, 38, 39, 40, 41, 42, 43, 44, 45, 46, 47, 48, 49] in the systematic review, involving a total of 483 participants. The studies encompassed participants with varying PA levels, including both physically active [37, 45, 46, 47] and physically inactive individuals [10, 38, 39, 40, 41, 42, 43, 44, 48, 49]. The intervention durations ranged from 4 to 12 weeks. The duration of each ExSn session varied across the studies, with eight studies employing sessions lasting no more than 2 min [37, 39, 40, 45, 46, 47, 48, 49] and six studies employing sessions lasting more than 2 min [10, 38, 41, 42, 43, 44]. The ExSn forms employed in the studies included sprint‐cycle in five studies [37, 44, 45, 46, 47], stair‐climbing in six studies [38, 39, 40, 42, 48, 49], and strength‐based exercises in three studies [10, 41, 43].

**TABLE 1:** Basic information from included studies.

Author	Year	Country	Participants	*n*	*n* (female)	Mean age (years)	ExSn method	Duration of per ExSn session
Allemeier et al.	1994	United States	Healthy men	17	N/A	IG (*N* = 11): 22.7 ± 5.0 years, CG (*N* = 6): 24.0 ± 2.3 years	Sprint‐cycle	30 s
Andersen et al.	2013	Denmark	Office workers	160	125	IG (*N* = 106): 42 ± 10 years, CG (*N* = 54): 43 ± 11 years	Stair‐walks	10 min
Boreham et al.	2000	United Kingdom	Sedentary but healthy female students	22	22	IG (*N* = 12): 19.8 ± 0.3 years, CG (*N* = 10): 20.3 ± 0.3 years	Stair‐climbing	2 min
Boreham et al.	2005	United Kingdom	Sedentary young women	15	15	IG (*N* = 8): 18.9 ± 0.6 years, CG (*N* = 7): 18.7 ± 0.8 years	Stair‐climbing	2 min
Brandt et al.	2024	United Kingdom	Female office workers	26	26	IG (*N* = 12): 42.1 ± 11.1 years, CG (*N* = 14): 49.9 ± 9.7 years	Resistance exercise snacking	10 min
Jenkins et al.	2019	Canada	Sedentary young adults	24	19	IG (*N* = 12): 20.0 ± 1.8 years, CG (*N* = 12): 19.3 ± 1.6 years	Stair‐climbing	4 min
Liang et al.	2022	United Kingdom	Self‐isolating older adults	32	19	IG (*N* = 15): 71.1 ± 3.6 years, CG (*N* = 17): 71.9 ± 4.7 years	Resistance exercise snacking	10 min
Metcalfe et al.	2012	United Kingdom	Sedentary but healthy young men	29	16	IG (*N* = 15): Male (*N* = 7): 26 ± 3, female (*N* = 8): 24 ± 3 CG (*N* = 14): Male (*N* = 6): 19 ± 1, female (*N* = 8): 21 ± 1	Sprint‐cycle	10 min
Perkin et al.	2019	United Kingdom	Healthy older adults	20	14	IG (*N* = 10): 70 ± 4 years CG (*N* = 10): 74 ± 5 years	Resistance exercise	10 min
Songsorn et al.	2016	United Kingdom	Healthy, sedentary or recreationally active participants	30	20	24 ± 6 years	Sprint‐cycle	20 s
Wong et al.	2024	Singapore	Physically active young adults	19	11	G (*N* = 11): 24.0 ± 2.8 years, CG (*N* = 8): 24.6 ± 1.7 years	Sprint‐cycle	30 s
Wu et al.	2020	Singapore	Recreationally active healthy adults	33	17	IG (*N* = 16): 34.1 ± 6.3 years, CG (*N* = 17): 35.1 ± 6.9 years	Sprint‐cycle	2 min
Yin et al.	2024	China	Inactive adults	29	15	IG (*N* = 14): 22.1 ± 2.1 years, CG (*N* = 15): 21.8 ± 3.0 years	Stair‐climbing	30 s
Zhou et al.	2025	China	Sedentary obese adults	27	14	IG (*N* = 14): 22.14 ± 1.88 years, CG (*N* = 13): 21.08 ± 1.32 years	Stair‐climbing	22.7 ± 9.8 s

Abbreviations: CG, control group; IG, intervention group.

### 3.3 Results of Data Synthesis

#### 3.3.1 Effects on Cardiorespiratory Fitness

**FIGURE 2:**
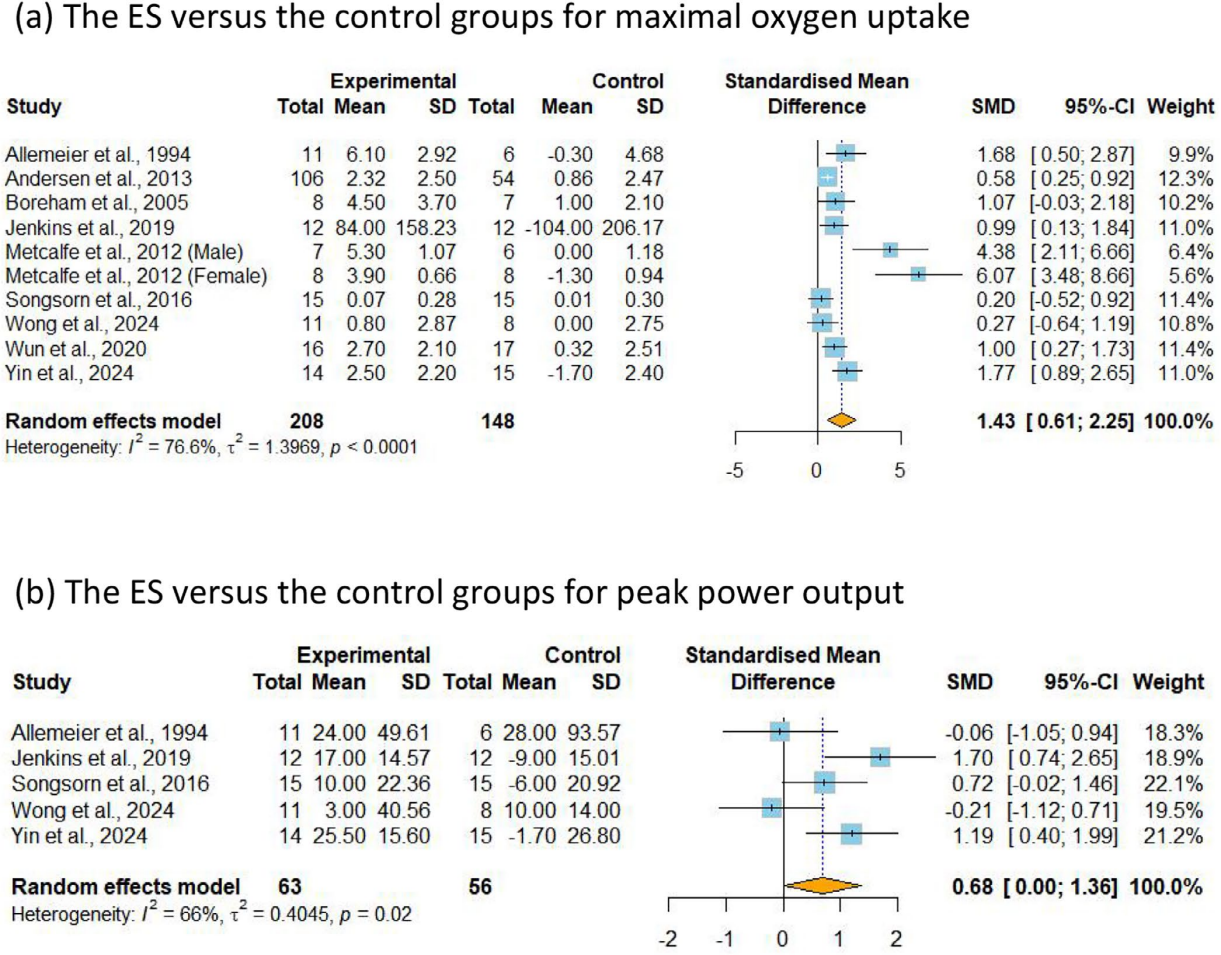
Meta‐analysis of the effects of ExSn on cardiorespiratory fitness. SMD (standardized mean difference) indicates the standard mean difference in the change values of the ExSn versus the control groups for (a) maximal oxygen uptake and (b) peak power output.

VO_2_max, an important indicator of cardiorespiratory fitness [50], was included in 10 studies [13, 37, 38, 39, 40, 44, 45, 46, 47, 48] with very low certainty of evidence, encompassing 378 participants. The initial analysis showed no significant differences in VO_2_max between the ExSn and control groups (SMD = 0.91, 95% CI −0.95 to 2.78, *p* = 0.336, *I* ^2^ = 87.3%). However, a sensitivity analysis was conducted by excluding a study with a high risk of bias [39], including 356 participants, which reduced heterogeneity. The revised analysis showed that ExSn can significantly improve VO_2_max compared to the control group (SMD = 1.43, 95% CI 0.61–2.25, *p* < 0.001, *I* ^2^ = 76.6%) (Figure 2a). Regarding the subgroup analysis, the improvement in VO_2_max appeared to be larger in physically inactive adults compared to physically active adults; however, no significant between‐group difference was observed (Table S3). This may be attributed to the small sample size and the limited number of studies included in the analysis. The analysis for PPO included a total of five studies [13, 37, 45, 46, 48], including 119 participants, with moderate certainty of evidence. The results showed that ExSn can significantly enhance PPO compared to the control groups (SMD = 0.68, 95% CI 0.00–1.36, *p* = 0.050, *I* ^2^ = 65.7%) (Figure 2b). Subgroup analyses based on participants' PA levels and the duration of each ExSn bout revealed significant group differences (*p* < 0.05) (Tables S3 and S4). Specifically, ExSn showed significant enhancement in PPO only in physically inactive adults and when the duration of each ExSn bout was more than 2 min.

#### 3.3.2 Effects on Lipid Profile

**FIGURE 3:**
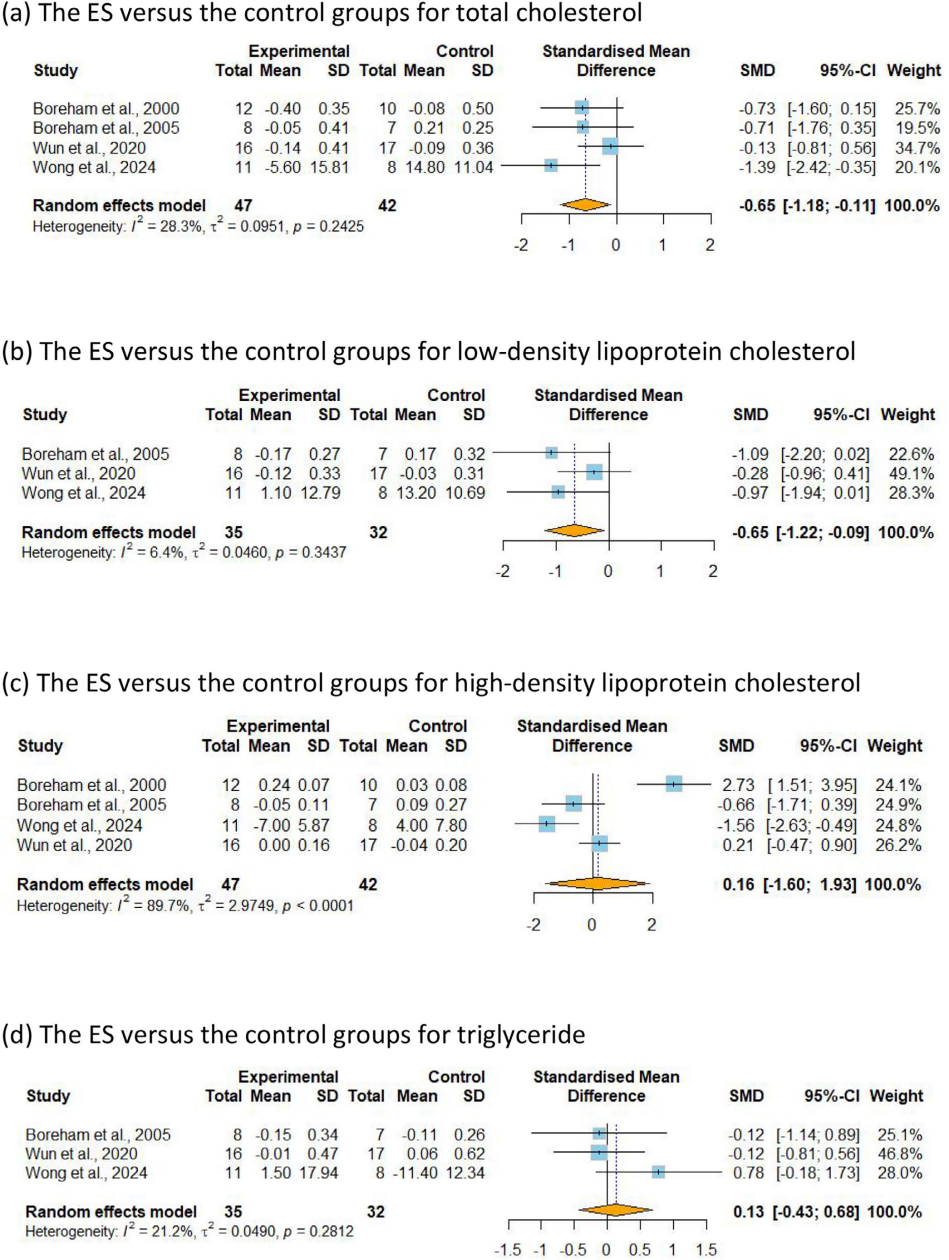
Meta‐analysis of the effects of ExSn on lipid profile. SMD indicates the standard mean difference in the change values of the ExSn versus the control groups for (a) total cholesterol, (b) low‐density lipoprotein cholesterol, (c) high‐density lipoprotein cholesterol, and (d) triglyceride.

TC has been included in four studies [39, 40, 46, 47] with low certainty of evidence, including 89 participants. The analysis results showed that participants assigned to the ExSn groups significantly reduced TC compared to the control groups (SMD = −0.65, 95% CI −1.18 to −0.11, *p* = 0.018, *I* ^2^ = 28.3%) (Figure 3a). Subgroup analyses revealed that only physically inactive adults showed a significant reduction in TC (SMD = −0.72, 95% CI −1.39 to −0.05, *p* = 0.036, *I* ^2^ = 0.0%) (Table S3), but no significant differences were observed between subgroups. Three studies [40, 46, 47] analyzed LDL‐C as an outcome, including 67 participants with moderate certainty of evidence. It was observed that ExSn showed a significant decrease in LDL‐C (SMD = −0.65, 95% CI −1.22 to −0.09, *p* = 0.023, *I* ^2^ = 6.4%) (Figure 3b), but subgroup analysis found no significant differences (Tables S3 and S4). Four RCTs [39, 40, 46, 47] with 89 participants analyzed HDL‐C as an outcome. There was no significant difference in HDL‐C with low certainty of evidence (SMD = 0.16, 95% CI −1.60 to 1.93, *p* = 0.856, *I* ^2^ = 89.7%) (Figure 3c). Three studies [40, 46, 47] with 72 participants analyzed TG as an outcome, and no significant differences were observed between the two groups (SMD = 0.13, 95% CI −0.43 to 0.68, *p* = 0.649, *I* ^2^ = 21.2%) (Figure 3d).

#### 3.3.3 Effects on Body Composition

**FIGURE 4:**
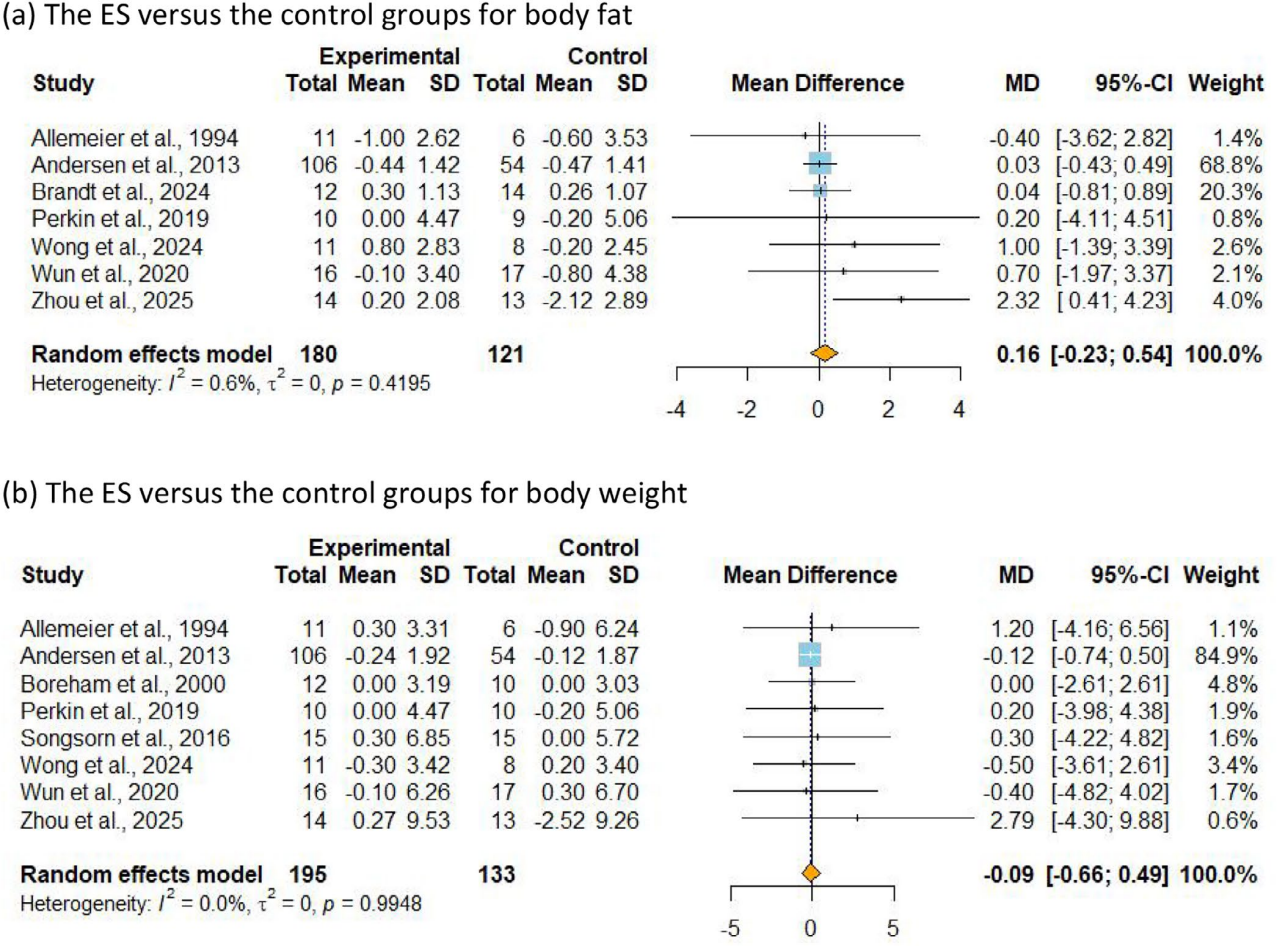
Meta‐analysis of the effects of ExSn on body composition. MD (mean difference) indicates the mean difference in the change values of the ExSn versus the control groups for (a) body fat and (b) body weight.

Seven studies [10, 37, 38, 41, 46, 47, 49], including 301 participants, analyzed %BF as an outcome. The meta‐analysis results indicated no significant difference in %BF between the ExSn and control groups (MD = 0.16%, 95% CI −0.22 to 0.54, *p* = 0.419, *I* ^2^ = 0.6%), with low certainty of evidence (Figure 4a). Subgroup analyses also showed no significant differences (Tables S3 and S4). BW data were available from eight studies [10, 37, 38, 39, 45, 46, 47, 49], including 328 participants, also with low certainty of evidence. The analysis revealed that ExSn did not result in a significant reduction in BW (MD = −0.08 kg, 95% CI −0.66 to 0.48, *p* = 0.769, *I* ^2^ = 0.0%) (Figure 4b), and subgroup analysis similarly found no significant differences (Tables S3 and S4).

#### 3.3.4 Subgroup Analysis

Detailed results of the subgroup analysis are provided in Tables S3 and S4 and Figures S2–S13, which present SMD/MD with corresponding 95% CIs for each subgroup separately. Significant group differences were observed only in PPO based on participants' physical activity levels and the duration of each ExSn bout.

### 3.4 Risk of Bias in Studies

The risk of bias was assessed using the RoB 2 tool for RCTs and the ROBINS‐I tool for non‐RCTs. A summary of the assessments is provided in Tables S7 and S8 in the Appendix S1. Among the RCTs, two studies had a high risk of bias, while ten had some concerns, primarily due to limitations in reporting and methodological issues. Among the non‐RCTs, one study had a serious risk of bias due to baseline confounding and missing data, while the other had a moderate risk of bias due to unmonitored external factors and lack of blinding.

### 3.5 Certainty of Evidence

The overall certainty of evidence was assessed using the GRADE tool and is presented in Table S2. The certainty of evidence was downgraded to low for the following outcomes: BW, %BF, TC, and HDL‐C. The certainty for PPO, LDL‐C, and TG was downgraded to moderate. VO_2_max was downgraded to very low certainty of evidence.

### 3.6 Publication Bias

We evaluated publication bias to determine the potential influence of selective publication on the results of our meta‐analysis. For VO_2_max, which met the minimum requirement of including at least ten studies, we performed a publication bias assessment (Figure S1). Egger's linear regression test was used to examine funnel plot asymmetry, and the results indicated no evidence of publication bias for VO_2_max (*p* = 0.628).

### 3.7 Meta‐Regression

Meta‐regression results showed that age was marginally associated with peak power output (*β* = −0.3482, *p* = 0.0858); while the duration of each bout and the duration of the trial were not significant predictors. For maximal oxygen uptake (VO_2_max), no significant associations were observed for age, the duration of each bout, or the duration of the trial (Table S6, Figures S14–S19).

## 4 Discussion

To the best of our knowledge, this is the first systematic review and meta‐analysis to investigate the effects of ExSn on cardiometabolic health and body composition in adults, focusing on both physically active and inactive individuals. Our findings suggest that ExSn can significantly improve cardiometabolic health by enhancing VO_2_max and PPO and decreasing TC and LDL‐C. Notably, the benefits of ExSn on cardiometabolic health are more pronounced in physically inactive adults than in physically active adults. Additionally, ExSn sessions lasting more than 2 min are more effective in improving cardiorespiratory fitness.

### 4.1 Effects of ExSn on Cardiorespiratory Fitness

Based on the results of our meta‐analysis, incorporating ExSn into adults' daily routines appears to be an effective strategy for improving cardiorespiratory fitness. Specifically, ExSn significantly enhanced VO_2_max (*p* < 0.001) and PPO (*p* = 0.05), both of which are important indicators of cardiorespiratory fitness [50, 51]. These findings are consistent with a previous systematic review, which also reported significant improvements in VO_2_max through even short bouts of high‐intensity interval training (≤ 5 min) (≤ 4 weeks) compared to control groups [52]. Previous studies indicate that short bursts of high‐intensity activity stimulate the release of growth factors and hormones, such as human growth hormone (HGH) and insulin‐like growth factor 1 (IGF‐1), which promote cardiovascular function and muscular adaptation [53, 54, 55]. These adaptations may include increased muscle hypertrophy, improved strength and power, enhanced aerobic and anaerobic capacity, and better cardiovascular efficiency, depending on the specific training components of ExSn [56, 57, 58].

Furthermore, the subgroup analysis based on the PA levels of participants revealed significant between‐group differences in PPO performance, with physically inactive adults benefiting the most from ExSn. Besides lower baseline values, another possible reason for the greater improvement in the inactive group could be their higher potential for adaptation and response to exercise interventions [59]. Physically inactive individuals often experience more pronounced physiological adaptations when starting a new exercise intervention, such as greater improvement in muscle strength and cardiovascular efficiency, compared to those who are already physically active [59, 60, 61, 62]. Although no significant subgroup difference in VO_2_max, ExSn still showed more notable improvements in VO_2_max compared to control groups, particularly among physically inactive adults. These findings suggest that ExSn can be particularly beneficial for those who are less physically active, such as sedentary office workers, potentially providing a practical and accessible means to enhance cardiorespiratory fitness. The results of the subgroup analysis based on the duration of the ExSn bout showed that when each bout lasted more than 2 min, there was a more prominent improvement in PPO. However, it is important to consider the feasibility of implementing such interventions, especially for office workers with limited time for physical activity who find traditional exercise less feasible. Future research should further explore the long‐term effects of ExSn and determine the optimal duration for each activity bout.

### 4.2 Effects of ExSn on Lipid Profile

In terms of lipid profile, the current meta‐analysis revealed that implementing ExSn did not significantly impact TG and HDL‐C levels. However, ExSn significantly decreased TC (*p* = 0.018) and LDL‐C (*p* = 0.023) compared to the control group. Regarding the subgroup analyses, different durations of each ExSn bout showed no significant difference in influencing lipid profiles. However, the subgroup analysis based on the PA levels of participants revealed that ExSn significantly decreased TC only in physically inactive adults. This more pronounced decrease in TC levels in physically inactive subjects can be attributed to their lower baseline physical activity levels, which means they have a higher potential for improvement and more significant metabolic adaptations when they begin exercising, compared to physically active subjects who may already have better metabolic profiles [63, 64, 65]. Additionally, although no significant between‐group differences were observed in HDL‐C (*p* = 0.96), there was an increasing trend in HDL‐C levels among physically inactive adults.

These findings suggest that while ExSn may not affect TG and HDL‐C levels, it can positively influence other aspects of the lipid profile, particularly in individuals who are physically inactive. These observations may be attributed to the influence of physical activity on lipid metabolism. Regular high‐intensity activities, even in short bursts, can enhance lipid metabolism by increasing the activity of lipoprotein lipase, an enzyme that plays a key role in breaking down triglycerides and promoting the clearance of LDL‐C from the bloodstream [66, 67, 68, 69]. Furthermore, previous research has shown that ExSn can improve insulin sensitivity and reduce insulin resistance [12, 70], which helps regulate lipid metabolism. Specifically, for individuals engaged in prolonged sitting with low PA levels, the incorporation of short bouts of physical activity through sitting interruption has demonstrated positive effects on blood glucose control and insulin levels [70, 71]. Enhanced insulin sensitivity increases the uptake of glucose by muscle cells, thereby reducing the liver's need to produce glucose through glycogenolysis and gluconeogenesis [72]. This reduction in glucose production by the liver can lead to lower blood glucose levels and subsequently reduce cholesterol synthesis, as high blood glucose levels are associated with increased cholesterol production [73]. Additionally, improved insulin sensitivity promotes the use of fatty acids as an energy source, decreasing the availability of free fatty acids in the bloodstream [66, 74]. These combined effects may help to lower TC and LDL‐C levels, contributing to a healthier lipid profile and improved cardiovascular health. Further research is warranted to better understand the effects of ExSn on lipid profile and to explore potential mechanisms underlying the observed associations.

### 4.3 Effects of ExSn on Body Composition

Our analysis revealed that the implementation of ExSn did not impact %BF and BW compared to the control group. Our findings align with a previous systematic review that has shown that short‐duration, high‐intensity exercise interventions are inefficient for the optimization of body composition [75]. While some studies suggest potential benefits in reducing %BF and BW [66, 76, 77], our meta‐analysis indicates that these effects may not be significant. The lack of significant changes in %BF and BW could be attributed to the relatively short duration and varying intensity of the ExSn interventions across the included studies (4–10 weeks). Typically, ExSn sessions last no more than 10 min, and this limited exercise volume may be insufficient to achieve meaningful fat loss. However, ExSn can still contribute to weight management by increasing overall physical activity, reducing sedentary time, and promoting healthy exercise habits [9]. Additionally, ExSn can improve insulin sensitivity and enhance lipid metabolism [12], which may indirectly support body composition improvements over the long term. Therefore, while the direct impact of ExSn on %BF and BW may be limited, their role in fostering positive health behaviors and metabolic benefits should not be overlooked.

### 4.4 Methodological Considerations During ExSn Implementation

Currently, the concept of ExSn is still relatively broad and lacks a standardized definition and application. Existing research on ExSn has primarily focused on assessing feasibility rather than establishing a precise definition [14]. Despite the varied concepts of ExSn, it is important to address several methodological considerations to ensure the validity of the findings and the effectiveness of the intervention. Firstly, unlike other forms of exercise, ExSn sessions typically last no more than 10 min and can be distributed throughout the day, which makes it convenient and has high compliance rates. Secondly, the intensity and type of exercise performed can also greatly influence results. High‐intensity interval training is commonly used, as it effectively ensures a high training intensity within a short period. Standardizing the intensity and type of exercises, or at least clearly reporting these variables, is essential for comparability and reproducibility. Additionally, while ExSn has demonstrated benefits for cardiometabolic health and lipid profiles, it is unlikely that physically inactive individuals will meet the recommended physical activity levels solely through this approach. Therefore, ExSn should be considered a complementary or alternative strategy for improving health in individuals unable to meet traditional physical activity guidelines, rather than a replacement for these recommendations. Lastly, short‐term studies may not capture the full impact of ExSn on body composition and health outcomes. Long‐term follow‐up is necessary to evaluate the sustainability of benefits and potential delayed effects.

### 4.5 Limitations

While our meta‐analysis has provided valuable insights, it is crucial to recognize certain limitations associated with our study. Firstly, there was notable inconsistency in the types of exercises employed across the included studies, such as sprint‐cycle, stair‐climbing, and strength‐based exercises. This heterogeneity in exercise modalities may have influenced the interpretation of the results and introduced additional variability. To address these limitations, future studies should consider comparing the effects of different exercise modalities and try to explore the most effective forms of exercise. Additionally, the certainty of evidence for maximal oxygen uptake in this meta‐analysis is very low, highlighting the need for further high‐quality studies to confirm these findings. Moreover, subgroup analyses for BMI, gender, and exercise snack frequency were not conducted due to limited reporting in the included studies. Future research should provide detailed information on these factors and investigate varying durations and frequencies of exercise snacks to determine optimal protocols for improving health outcomes.

## 5 Conclusion

Our systematic review and meta‐analysis provide promising evidence that supports the effectiveness of incorporating ExSn into daily routines for enhancing cardiometabolic health in adults, with a particular emphasis on physically inactive individuals. These findings have important implications for healthcare practitioners and public health professionals in promoting physical activity and improving overall health outcomes. However, future research should explore the long‐term effects of exercise snacks; determine optimal duration and frequency; and identify the most effective forms of exercise within this approach.

## Author Contributions

Ke‐wen Wan assumed the lead role in this study, overseeing data access, systematic search for eligible studies, data extraction, result interpretation, manuscript drafting, and serving as the guarantor. Zi‐han Dai, Po‐san Wong, Evander Fung‐chau Lei, and Bjorn T. Tam actively participated in the study's conception, design, systematic search for eligible studies, data extraction, and manuscript composition. Bjorn T. Tam, Wendy Y. Huang, Jonathan P. Little, and Feng‐Chang Lin played a key role in drafting the manuscript and providing critical revisions. Bjorn T. Tam provided overall supervision and strategic direction for the study, and managed funding acquisition. All authors thoroughly reviewed and granted approval for the final manuscript submission.

## Ethics Statement

The authors have nothing to report.

## Consent

The authors have nothing to report.

## Conflicts of Interest

The authors declare no conflicts of interest.

## Supporting information


**Table S1:** Baseline characteristics of the included studies.
**Table S2:** Recommendation, assessment, development and evaluation tool for the assessment of certainty of evidence.
**Table S3:** Subgroup analysis according to the physical activity level of participants.
**Table S4:** Subgroup analysis according to the duration for each bout of ExSn.
**Table S5:** Searching strategy.
**Table S6:** Meta‐regression of moderators of effects on ExSn on cardiometabolic health.
**Table S7:** Risk of bias assessment for all outcome categories.
**Table S8:** Summary of risk of bias (RoB) assessments for studies using ROBINS‐I.
**Figure S1:** Funnel plot for publication bias detection on maximal oxygen uptake. The funnel plot shows the observed standardized mean differences (on the x‐axis) against standard errors (on the y‐axis).
**Figure S2:** Meta‐analysis of ExSn versus controls on body fat, using mean difference (MD) to indicate the difference in change values between ExSn and control groups. Subgroup analysis based on participants' physical activity level (active vs. inactive).
**Figure S3:** Meta‐analysis of ExSn versus controls on body weight, using mean difference (MD) to indicate the difference in change values between ExSn and control groups. Subgroup analysis based on participants' physical activity level (active vs. inactive).
**Figure S4:** Meta‐analysis of ExSn versus controls on maximal oxygen uptake, using SMD to indicate the difference in change values between ExSn and control groups. Subgroup analysis based on participants' physical activity level (active vs. inactive).
**Figure S5:** Meta‐analysis of ExSn versus controls on peak power output, using SMD to indicate the difference in change values between ExSn and control groups. Subgroup analysis based on participants' physical activity level (active vs. inactive).
**Figure S6:** Meta‐analysis of ExSn versus controls on total cholesterol, using SMD to indicate the difference in change values between ExSn and control groups. Subgroup analysis based on participants' physical activity level (active vs. inactive).
**Figure S7:** Meta‐analysis of ExSn versus controls on low‐density lipoprotein cholesterol, using SMD to indicate the difference in change values between ExSn and control groups. Subgroup analysis based on participants' physical activity level (active vs. inactive).
**Figure S8:** Meta‐analysis of ExSn versus controls on high‐density lipoprotein cholesterol, using SMD to indicate the difference in change values between ExSn and control groups. Subgroup analysis based on participants' physical activity level (active vs. inactive).
**Figure S9:** Meta‐analysis of ExSn versus controls on triglycerides, using SMD to indicate the difference in change values between ExSn and control groups. Subgroup analysis based on participants' physical activity level (active vs. inactive).
**Figure S10:** Meta‐analysis of ExSn versus controls on body fat, using MD to indicate the difference in change values between ExSn and control groups. Subgroup analysis based on the duration for each bout of ExSn (≤ 2 min vs. > 2 min).
**Figure S11:** Meta‐analysis of ExSn versus controls on body weight, using MD to indicate the difference in change values between ExSn and control groups. Subgroup analysis based on the duration for each bout of ExSn (≤ 2 min vs. > 2 min).
**Figure S12:** Meta‐analysis of ExSn versus controls on peak power output, using SMD to indicate the difference in change values between ExSn and control groups. Subgroup analysis based on the duration for each bout of ExSn (≤ 2 min vs. > 2 min).
**Figure S13:** Meta‐analysis of ExSn versus controls on maximal oxygen uptake, using SMD to indicate the difference in change values between ExSn and control groups. Subgroup analysis based on the duration for each bout of ExSn (≤ 2 min vs. > 2 min).
**Figure S14:** Meta‐regression of peak power output moderated by age.
**Figure S15:** Meta‐regression of peak power output moderated by the duration of each ExSn bout.
**Figure S16:** Meta‐regression of peak power output moderated by the duration of the trial.
**Figure S17:** Meta‐regression of maximal oxygen uptake moderated by age.
**Figure S18:** Meta‐regression of maximal oxygen uptake moderated by the duration of each ExSn bout.
**Figure S19:** Meta‐regression of maximal oxygen uptake moderated by the duration of the trial.

## Data Availability

The datasets used and/or analyzed during the current study are available from the corresponding author upon reasonable request.
