# Supplementary material for: Effects of Exercise Snacks on Cardiometabolic Health and Body Composition in Adults: A Systematic Review and Meta‐Analysis
Source: Scand J Med Sci Sports. 2025 Aug 14;35(8):e70114. doi: 10.1111/sms.70114 (PMC12354995; doi:10.1111/sms.70114)
Supplement: Supplementary file 1 — Table S1: Baseline characteristics of the included studies. Table S2: Recommendation, assessment, development and evaluation tool for the assessment of certainty of evidence. Table S3: Subgroup analysis according to the physical activity level of participants. Table S4: Subgroup analysis according to the duration for each bout of ExSn. Table S5: Searching strategy. Table S6: Meta‐regression of moderators of effects on ExSn on cardiometabolic health. Table S7: Risk of bias assessment for all outcome categories. Table S8: Summary of risk of bias (RoB) assessments for studies using ROBINS‐I. Figure S1: Funnel plot for publication bias detection on maximal oxygen uptake. The funnel plot shows the observed standardized mean differences (on the x‐axis) against standard errors (on the y‐axis). Figure S2: Meta‐analysis of ExSn versus controls on body fat, using mean difference (MD) to indicate the difference in change values between ExSn and control groups. Subgroup analysis based on participants' physical activity level (active vs. inactive). Figure S3: Meta‐analysis of ExSn versus controls on body weight, using mean difference (MD) to indicate the difference in change values between ExSn and control groups. Subgroup analysis based on participants' physical activity level (active vs. inactive). Figure S4: Meta‐analysis of ExSn versus controls on maximal oxygen uptake, using SMD to indicate the difference in change values between ExSn and control groups. Subgroup analysis based on participants' physical activity level (active vs. inactive). Figure S5: Meta‐analysis of ExSn versus controls on peak power output, using SMD to indicate the difference in change values between ExSn and control groups. Subgroup analysis based on participants' physical activity level (active vs. inactive). Figure S6: Meta‐analysis of ExSn versus controls on total cholesterol, using SMD to indicate the difference in change values between ExSn and control groups. Subgroup analysis based on participan [file SMS-35-e70114-s001.pdf]

Supplementary Information

Effects of Exercise Snacks on Cardiometabolic Health and Body Composition in Adults: A Systematic Review and Meta-analysis

Author Block: Ke-wen Wan[1,2], Zi-han Dai[3], Po-san Wong[3], Wendy Ya-jun Huang[1,2], Evander Fung-chau Lei[1,2], Jonathan P. Little[4], Feng-Chang Lin[5], *Bjorn T. Tam[1,2].

1 Academy of Wellness and Human Development, Faculty of Arts and Social Sciences, Hong Kong

Baptist University, Kowloon Tong, Hong Kong, China

2 Dr. Stephen Hui Research Centre for Physical Recreation and Wellness, Hong Kong Baptist University, Kowloon Tong, Hong Kong, China

3 Department of Sports Science and Physical Education, The Chinese University of Hong Kong, Hong Kong, China

4 School of Health and Exercise Sciences, University of British Columbia, Kelowna, BC, Canada

5 Department of Orthopaedic Surgery, University of North Carolina School of Medicine, Chapel Hill, NC, USA

Correspondence: Bjorn T. Tam (bjorntam@hkbu.edu.hk)

Table S1 Characteristic of included studies

Author	Country	Participants Health status, N (sex), age	Study Design	Study duration	Interventions description	Duration of per session	Main outcomes	Main findings
Allemeier et al., 1994	United States	Healthy men, N = 17; IG (N = 11): 22.7 ± 5.0y, CG (N = 6): 24.0 ± 2.3y.	RCT	6 weeks	Sprint-cycle, 30-s supramaximal sprints for three consecutive bouts with 20 minutes of rest between bouts, 2-3 times per week.	30 seconds	Peak power, Maximal oxygen uptake.	IG v.s. CG: NS.
Andersen et al., 2013	Denmark	Office workers, N = 160 (35 males); IG (N = 106): 42 ± 10y, CG (N = 54): 43 ± 11y.	RCT	10 weeks	Stair-walks, 10 minutes a day during working days (5 days a week)	10 minutes	Oxygen uptake, Body weight, Fat percentage.	IG v.s. CG: Aerobic fitness (oxygen uptake) ↑

Boreham et al., 2000	United Kingdom	Sedentary but healthy female students, N = 22;<br>IG (N = 12): 19.8 ± 0.3y,<br>CG (N = 10): 20.3 ± 0.3y.	RCT	7 weeks	Stair-climbing, increased stair ascents from 1 per day in week 1 to 6 per day in weeks 6-7, using a 199-step public staircase at a pace of 135 s/ascent (88 steps/min).	2 minutes	Body mass,<br>Total cholesterol,<br>High-density lipoprotein cholesterol,<br>Mean oxygen uptake.	IG v.s. CG:<br>Total cholesterol ↑<br>High-density lipoprotein cholesterol ↑<br>Mean oxygen uptake ↑
Boreham et al., 2005	United Kingdom	Sedentary young women, N = 15;<br>IG (N = 8): 18.9 ± 0.6y,<br>CG (N = 7): 18.7 ± 0.8y.	RCT	8 weeks	Stair-climbing, increased from 1 ascent/day in week 1 to 5 ascents/day in weeks 7-8. 5 days/week on a 199-step public staircase (2 minutes), at a stepping rate of 90 steps/minute.	2 minutes	Maximal oxygen consumption,<br>Total cholesterol,<br>High-density lipoprotein cholesterol,<br>Low-density lipoprotein cholesterol,<br>Triglycerides.	IG v.s. CG:<br>Maximal oxygen consumption ↑<br>Low-density lipoprotein cholesterol ↑

Brandt et al., 2024	United Kingdom	Female office workers (mainly sitting or standing job), N = 26; IG (N = 12): 42.1 ± 11.1y, CG (N = 14): 49.9 ± 9.7y.	Non-RCT	12 weeks	Resistance exercise snacking, performed a 10-min resistance exercise snacking on 5 days per weeks. Each session included 30-60 seconds of consecutive strength and mobility exercises.	10 minutes	Cardiovascular fitness, Body fat.	IG v.s. CG: Muscle mass ↑
Jenkins et al., 2019	Canada	Sedentary young adults, N = 24 (5 males); IG (N = 12): 20.0 ± 1.8y, CG (N = 12): 19.3 ± 1.6y.	RCT	6 weeks	Stair-climbing, 3 bouts/day of vigorously ascending a 3-flight stairwell (60 steps), separated by 1–4 h of recovery.	4 minutes	Absolute peak oxygen uptake, Peak power output.	IG v.s. CG: Absolute peak oxygen uptake ↑ Peak power output ↑
Liang et al., 2022	United Kingdom	Self-isolating older adults, N = 32 (13 males); IG (N = 15): 71.1 ± 3.6y, CG (N = 17): 71.9 ± 4.7y.	RCT	4 weeks	Five movements, each undertaken for 1 minute with the aim of completing as many repetitions as possible. Participants	10 minutes	NA	IG & CG: Physical function ↑ IG v.s. CG: Adherence ↑

					rested for 1 min between exercises.			
Metcalfe et al., 2012	United Kingdom	Sedentary but healthy young men, N = 29 (13 males);<br>IG (N = 15):<br>Male (N = 7): 26 ± 3,<br>Female (N = 8): 24 ± 3;<br>CG (N = 14):<br>Male (N = 6): 19 ± 1;<br>Female (N = 8): 21 ± 1.	RCT	6 weeks	Sprint-cycle, progressively increased from 1 to 3 bouts×10-s to 20-s each session, 1 session per day, 3 days per week for 6 weeks.	10 minutes	Maximal oxygen uptake.	IG v.s. CG:<br>Maximal oxygen uptake ↑<br>Insulin sensitivity ↑
Perkin et al., 2019	United Kingdom	Healthy older adults, N = 20 (6 males);<br>IG (N = 10): 70 ± 4y,<br>CG (N = 10): 74 ± 5y.	RCT	28 days	Five movements, each undertaken for 1 minute with the aim of completing as many repetitions as possible, twice a day. Participants rested for 1 min between exercises.	10 minutes	Body mass,<br>Body fat.	IG v.s. CG:<br>Sit-to-stand scores ↑

Songsorn et al., 2016	United Kingdom	Healthy, sedentary or recreationally active participants, N = 30 (N = 10 males); 24 ± 6y.	RCT	4 weeks	Sprint-cycle, 1 bout×20-s each session, 1 session per day, 3 days per week for 4 weeks.	20 seconds	Body mass, Maximal power output, Maximal oxygen uptake.	IG v.s. CG: NS.
Wong et al., 2024	Singapore	Physically active young adults, N = 19 (8 males); IG (N = 11): 24.0 ± 2.8y, CG (N = 8): 24.6 ± 1.7y.	RCT	6 weeks	Sprint-cycle, one all-out bout of the 30-s per day (Mondays to Fridays).	30 seconds	Body mass, Body fat, High-density lipoprotein cholesterol, Low-density lipoprotein cholesterol, Total cholesterol, Triglycerides, Maximal oxygen uptake.	IG v.s. CG: NS.

Wun et al., 2020	Singapore	Recreationally active healthy adults, N = 33 (16 males); IG (N = 16): 34.1 ± 6.3y, CG (N = 17): 35.1 ± 6.9y.	Non-RCT	6 weeks	Sprint-cycle, 1 bout×30-s each session, 3 sessions per day, 3 days per week	2 minutes	Body mass, Body fat, Maximal oxygen uptake, High-density lipoprotein cholesterol, Low-density lipoprotein cholesterol, Total cholesterol, Triglycerides.	IG v.s. CG: Maximal oxygen uptake ↑ Leg strength ↑
Yin et al., 2024	China	Inactive adults, N = 29 (14 males); IG (N = 14): 22.1 ± 2.1y, CG (N = 15): 21.8 ± 3.0y.	RCT	6 weeks	Stair-climbing, complete 3 × 30 s all-out stair climbs in one day with ≥1 h rest between sessions, 3 days per week	30 seconds	Maximal oxygen uptake, Peak power.	IG v.s. CG: Maximal oxygen uptake ↑ Peak power ↑

Zhou et al., 2025	China	Sedentary obese adults, N = 27 (13 males);<br>IG (N = 14): 22.14 ± 1.88y,<br>CG (N = 13): 21.08 ± 1.32y.	RCT	12 weeks	Stair-climbing, complete 6 × 60-step stair climbs at a challenging self-selected pace in one day with ≥1 h rest between sessions, 4 days per week for 12 weeks.	22.7 ± 9.8 seconds	Body weight,<br>Body fat percentage.	IG v.s. CG: NS.

Note. CG, control group; IG, intervention group; RCT, randomized controlled trial.

Table S2 Recommendation, Assessment, Development and Evaluation tool for the assessment of certainty of evidence.

Outcome	Study design	Grade assessment					
		**Risk of bias**	**Inconsistency**	**Indirectness**	**Imprecision**	**Publication bias**	**Certainty of evidence**
Maximal oxygen uptake	10 RCTs	Serious [a]	Serious [b]	Not serious	Serious [c]	None	Very low
Peak power output	5 RCTs	Not serious	Not serious	Not serious	Serious [c]	None	Moderate
Body weight	8 RCTs	Serious [a]	Not serious	Not serious	Serious [c]	None	Low
Body fat	7 RCTs	Serious [a]	Not serious	Not serious	Serious [c]	None	Low
Total cholesterol	4 RCTs	Serious [a]	Not serious	Not serious	Serious [c]	None	Low
High-density lipoprotein cholesterol	4 RCTs	Serious [a]	Not serious	Not serious	Serious [c]	None	Low
Low-density lipoprotein cholesterol	3 RCTs	Not serious	Not serious	Not serious	Serious [c]	None	Moderate
Triglycerides	3 RCTs	Not serious	Not serious	Not serious	Serious [c]	None	Moderate

a. The included study showed the high risk of bias.
b. I^2 values showed high heterogeneity.
c. The sample size is no more than 400.
d. I^2 values showed substantial heterogeneity.

Table S3 Subgroup analysis according to the physical activity level of participants

Outcome	Physically active	MD/SMD	95%CI	I^2	Physically inactive	MD/SMD	95%CI	I^2	Subgroup differences
Body fat (%)	3	0.57	[-0.99, 2.13]	0.0%	4	0.13	[-0.26, 0.53]	0.0%	Chi^2= 0.29 (p=0.59)
Body weight (kg)	4	-0.07	[-2.11, 1.98]	0.0%	4	-0.09	[-0.68, 0.51]	0.0%	Chi^2= 0.00 (p=0.98)
HDL-C	2	-0.62	[-2.36, 1.11]	86.7%	2	1.02	[-2.30, 4.34]	94.1%	Chi^2= 0.00 (p=0.96)
LDL-C	2	-0.53	[-1.18, 0.12]	2.5%	1	-1.09	[-2.20, 0.02]	--	Chi^2= 0.72 (p=0.40)
PPO	3	0.21	[-0.41, 0.83]	30.0%	2	1.40	**[0.79, 2.01]**	0.0%	**Chi^2= 7.14 (p<0.01)**
TC	2	-0.69	[-1.92, 0.53]	74.8%	2	-0.72	**[-1.39, -0.05]**	0.0%	Chi^2= 0.00 (p=0.97)
TG	2	0.26	[-0.61, 1.13]	55.9%	1	-0.12	[-1.14, 0.89]	--	Chi^2= 0.32 (p=0.57)
VO2max	4	0.71	**[0.11, 1.31]**	50.0%	6	1.93	**[0.69, 3.18]**	81.2%	Chi^2= 3.03 (p=0.08)

Note. HDL-C, high-density lipoprotein cholesterol; LDL-C, low-density lipoprotein cholesterol; PPO, peak power output; TC, total cholesterol; TG, triglycerides; VO2max, maximal oxygen uptake.

Table S4 Subgroup analysis according to the duration for each bout of ExSn

Outcome	≤ 2 min	MD/SMD	95%CI	I^2	> 2 min	MD/SMD	95%CI	I^2	Subgroup differences
Body fat (%)	4	1.27	[0.06, 2.48]	0.0%	3	0.03	[-0.37, 0.44]	0.0%	Chi^2= 3.61 (p=0.06)
Body weight (kg)	6	-0.10	[-1.47, 1.67]	0.0%	2	-0.11	[-0.73, 0.50]	0.0%	Chi^2= 0.06 (p=0.81)
PPO	4	0.46	[-0.18, 1.10]	5.4%	1	1.14	**[0.74, 2.65]**	--	**Chi^2= 4.43 (p=0.03)**
VO2max	7	-0.16	[-2.52, 2.20]	88.3%	4	2.76	**[0.21, 5.31]**	89.0%	Chi^2= 2.71 (p=0.10)

Note. The subgroup analysis was conducted based on exercise duration (≤2 minutes vs. >2 minutes) because 2 minutes represented a common duration in the included studies, despite the inconsistent definitions of exercise snacks across the literature (e.g., no more than 1 minute, 1 minute, 2–5 minutes, or 2–10 minutes). This cutoff allowed for a meaningful comparison of studies while reflecting the variability in exercise snack interventions. PPO, peak power output; VO2max, maximal oxygen uptake.

Table S5 Searching strategy

Database name	Pubmed
Full search strategies	(("Exercise snack*" OR "Snacktivity" OR "Movement snack*" OR "Physical activity break*" OR "Movement break*" OR "Short bouts of exercise" OR "Physical activity short bouts" OR "Exercise short bouts" OR "Short bouts of stair climbing")[tiab]) OR (("exercise" OR "physical activity" OR "movement")[tiab] AND ("short bouts" OR "brief bouts" OR "intermittent bouts" OR "low-volume bouts" OR "mini bouts")[tiab]) NOT (animals[mh] NOT humans[mh])
Limits and restrictions	English
Dates of searches	22/05/2025
Hits	2366
**Database name**	**Web of Science**
Full search strategies	TS=("Exercise snack*" OR "Snacktivity" OR "Movement snack*" OR "Physical activity break*" OR "Movement break*" OR "Short bouts of exercise" OR<br>"Physical activity short bouts" OR "Exercise short bouts" OR "Short bouts of stair climbing")<br>OR TS=(("exercise" OR "physical activity" OR "movement") AND ("short*" OR "brief*" OR "intermittent*"))
Limits and restrictions	Language: English
Dates of searches	22/05/2025
Hits	1501
**Database name**	**Cochrane Central Register of Controlled Trials (CENTRAL)**
Full search strategies	("Exercise snack*" OR "Snacktivity" OR "Movement snack*" OR "Physical activity break*" OR "Movement break*" OR "Short bouts of exercise" OR "Physical activity short bouts" OR "Exercise short bouts" OR "Short bouts of stair climbing") in Title Abstract Keyword OR ("exercise" OR "physical activity" OR "movement") AND ("short bouts" OR "brief bouts" OR "intermittent bouts" OR "low-volume bouts" OR "mini bouts") in Title Abstract Keyword NOT "animal" OR "rat" OR "mice" OR "mouse" in Title Abstract Keyword - with Cochrane Library publication date Between Jan 2022 and Jan 2025, in Trials (Word variations have been searched)
Limits and restrictions	english language
Dates of searches	22/05/2025
Hits	117
**Database name**	**CINAHL**
Full search strategies	XB ("Exercise snack*" OR "Snacktivity" OR "Movement snack*" OR "Physical activity break*" OR "Movement break*" OR "Short bouts of exercise" OR "Physical activity short bouts" OR "Exercise short bouts" OR "Short bouts of stair climbing") OR XB (("exercise" OR "physical activity" OR "movement") AND ("short bouts" OR "brief bouts" OR "intermittent bouts" OR "low-volume bouts" OR "mini bouts"))
Limits and restrictions	full text and human and english language
Dates of searches	22/05/2025
Hits	281

Database name	Scopus
Full search strategies	TITLE-ABS-KEY (("Exercise snack*" OR "Snacktivity" OR "Movement snack*" OR "Physical activity break*" OR "Movement break*" OR "Short bouts of exercise" OR "Physical activity short bouts" OR "Exercise short bouts" OR "Short bouts of stair climbing")) OR TITLE-ABS-KEY (("exercise" OR "physical activity" OR "movement") AND ("short bouts" OR "brief bouts" OR "intermittent bouts" OR "low-volume bouts" OR "mini bouts")) AND NOT TITLE-ABS-KEY ("animal" OR "rat" OR "mice" OR "mouse") AND (LIMIT-TO (LANGUAGE , "English"))
Limits and restrictions	english language
Dates of searches	22/05/2025
Hits	904

Embase results:

#	Query	Results from 22 May 2025
1	"Exercise snack*".ab,ti.	58
2	"Snacktivity".ab,ti.	8
3	"Movement snack* ".ab,ti.	0
4	"Physical activity break* ".ab,ti.	106
5	"Movement break* ".ab,ti.	36
6	"Short bouts of exercise".ab,ti.	68
7	"Physical activity short bouts".ab,ti.	1
8	"Exercise short bouts".ab,ti.	1
9	"Short bouts of stair climbing".ab,ti.	3
10	1 or 2 or 3 or 4 or 5 or 6 or 7 or 8 or 9	275
11	"exercise".ab,ti.	469,167
12	"physical activity".ab,ti.	225,518
13	"movement".ab,ti.	382,975
14	"short bouts".ab,ti.	562
15	"brief bouts".ab,ti.	145
16	"intermittent bouts".ab,ti.	170
17	"low-volume bouts".ab,ti.	0
18	"mini bouts".ab,ti.	2
19	11 or 12 or 13	1,010,906
20	14 or 15 or 16 or 17 or 18	861
21	19 and 20	493
22	10 or 21	683
23	limit 22 to english language	674

Table S6 Meta-regression of moderators of effects on ExSn on cardiometabolic health

Moderator	Number of RCTs	β	95% CI	p-value	F-value	I^2
Peak power output						
Age	5	-0.3482	[-0.7870, 0.0906]	0.0858	6.376	23.56%
Duration of each bout (in minutes)	5	0.3412	[-0.3548, 1.0372]	0.2167	1.56	57.23%
Duration of trial (in weeks)	5	-0.0257	[-1.6221, 1.5707]	0.9624	-0.0512	74.73%
Maximal oxygen uptake						
Age	10	-0.0515	[-0.2141, 0.1111]	0.486	-0.7304	87.60%
Duration of each bout (in minutes)	10	0.2033	[-0.0685, 0.4751]	0.1228	1.7249	85.79%
Duration of trial (in weeks)	10	-0.0884	[-0.8798, 0.7030]	0.8032	-0.2576	88.54%

Note. *CI* confidence interval. ExSn, exercise snacks. RCT, randomized controlled trial.

Table S7 Risk of Bias Assessment for All Outcome Categories

Study	Risk of Bias Domain					
	**Randomization process**	**Deviations from intended interventions**	**Missing outcome data**	**Measurement of outcome**	**Selection of reported results**	**Overall risk of bias**
**Body composition**						
Allemeier et al., 1994	Some concerns	Some concerns	Low	Low	Some concerns	Some concerns
Andersen et al., 2013	Low	Some concerns	Low	Low	Low	Some concerns
Boreham et al., 2000	High	Some concerns	Low	Low	Some concerns	High
Songsorn et al., 2016	Some concerns	Some concerns	Low	Low	Some concerns	Some concerns
Wong et al., 2024	Some concerns	Some concerns	Low	Low	Low	Some concerns
Zhou et al., 2025	Some concerns	Some concerns	Low	Low	Low	Some concerns
**Cardiorespiratory fitness**						

Allemeier et al., 1994	Some concerns	Some concerns	Low	Low	Some concerns	Some concerns
Andersen et al., 2013	Low	Some concerns	Low	Low	Low	Some concerns
Boreham et al., 2005	Some concerns	Some concerns	Low	Low	Some concerns	Some concerns
Jenkins et al., 2019	High	Some concerns	Low	Low	Some concerns	High
Metcalfe et al., 2012	Some concerns	Some concerns	Low	Low	Some concerns	Some concerns
Songsorn et al., 2016	Some concerns	Some concerns	Low	Low	Some concerns	Some concerns
Wong et al., 2024	Some concerns	Some concerns	Low	Low	Low	Some concerns
Yin et al., 2024	Some concerns	Some concerns	Low	Low	Low	Some concerns
**Lipid profile**						
Boreham et al., 2000	High	Some concerns	Low	Low	Some concerns	High
Boreham et al., 2005	Some concerns	Some concerns	Low	Low	Some concerns	Some concerns
Wong et al., 2024	Some concerns	Some concerns	Low	Low	Low	Some concerns

Table S8 Summary of Risk of Bias (RoB) Assessments for Studies Using ROBINS-I

	**Risk of Bias Domain**							
**Study**	**Risk of bias due to confounding**	**Risk of bias in classification of interventions**	**Risk of bias in selection of participants into the study**	**Risk of bias due to deviations from intended interventions**	**Risk of bias due to missing data**	**Risk of bias arising from measurement of the outcome**	**Risk of bias in selection of the reported result**	**Overall Risk of Bias**
**Body composition**								
Brandt et al., 2024	Serious risk of bias	Moderate risk of bias	Low risk of bias	Moderate risk of bias	Serious risk of bias	Moderate risk of bias	Low risk of bias	Serious risk of bias
Wun et al., 2020	Moderate risk of bias	Moderate risk of bias	Low risk of bias	Moderate risk of bias	Low risk of bias	Moderate risk of bias	Low risk of bias	Moderate risk of bias

**Cardiorespiratory fitness**								
Wun et al., 2020	Moderate risk of bias	Moderate risk of bias	Low risk of bias	Moderate risk of bias	Low risk of bias	Moderate risk of bias	Low risk of bias	Moderate risk of bias
**Lipid profile**								
Wun et al., 2020	Moderate risk of bias	Moderate risk of bias	Low risk of bias	Moderate risk of bias	Low risk of bias	Moderate risk of bias	Low risk of bias	Moderate risk of bias

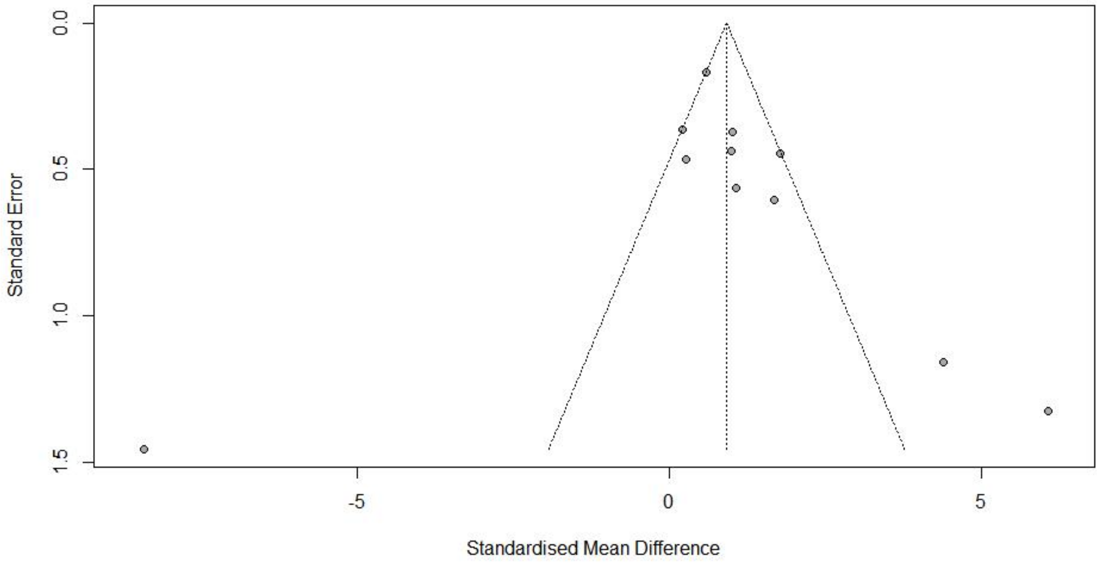

Figure S1 Funnel plot for publication bias detection on maximal oxygen uptake. The funnel plot shows the observed standardized mean differences (on the x-axis) against standard errors (on the y-axis).

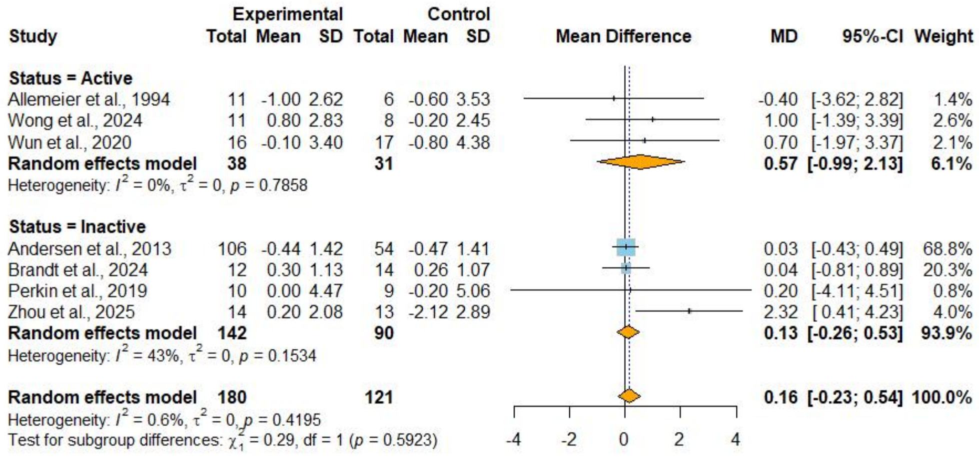

Figure S2 Meta-analysis of ExSn versus controls on body fat percentage, using mean difference (MD) to indicate the difference in change values between ExSn and control groups. Subgroup analysis based on participants' physical activity level (active vs. inactive).

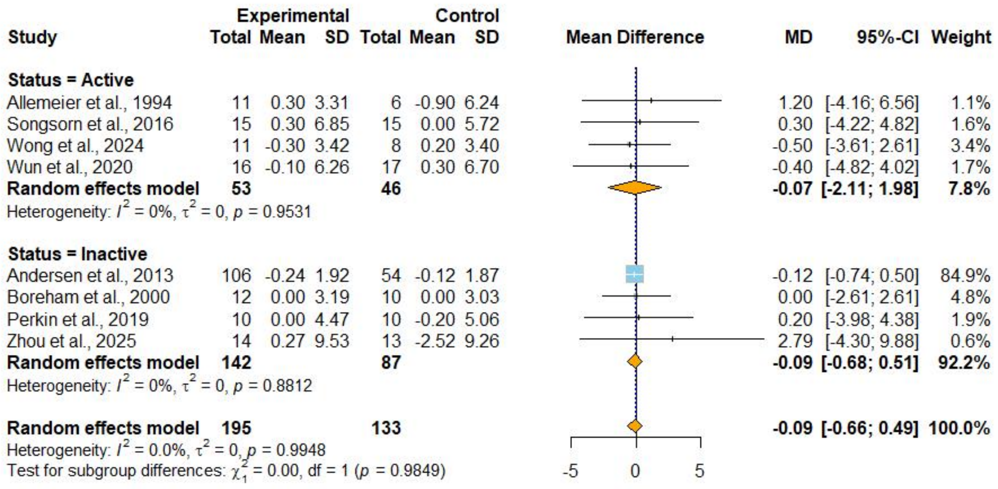

Figure S3 Meta-analysis of ExSn versus controls on body weight, using mean difference (MD) to indicate the difference in change values between ExSn and control groups. Subgroup analysis based on participants' physical activity level (active vs. inactive).

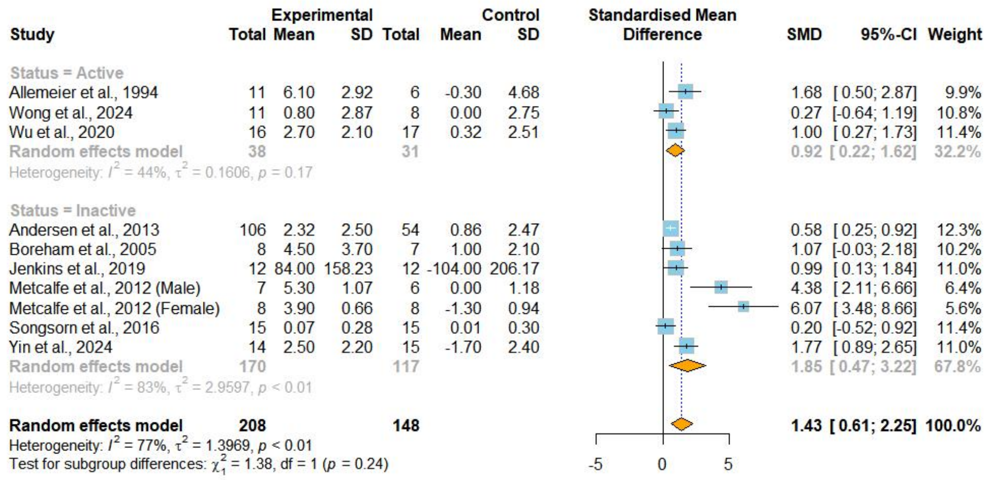

Figure S4 Meta-analysis of ExSn versus controls on maximal oxygen uptake, using SMD to indicate the difference in change values between ExSn and control groups. Subgroup analysis based on participants' physical activity level (active vs. inactive).

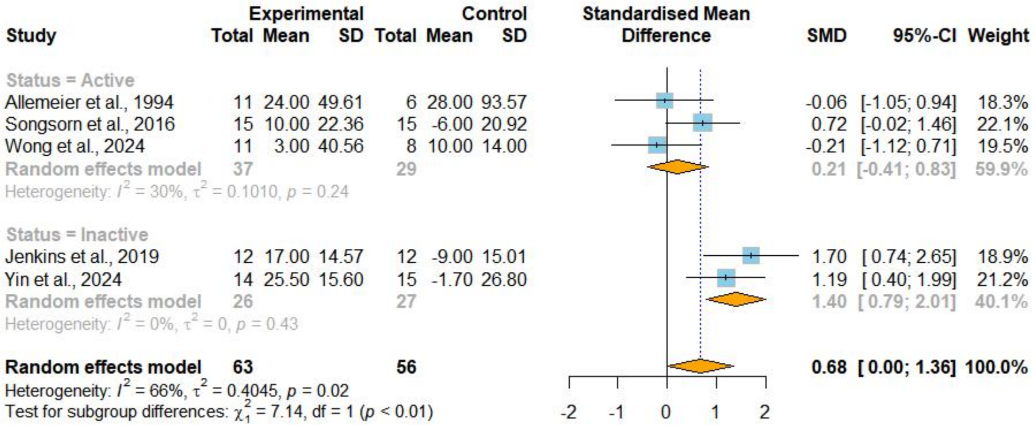

Figure S5 Meta-analysis of ExSn versus controls on peak power output, using SMD to indicate the difference in change values between ExSn and control groups. Subgroup analysis based on participants' physical activity level (active vs. inactive).

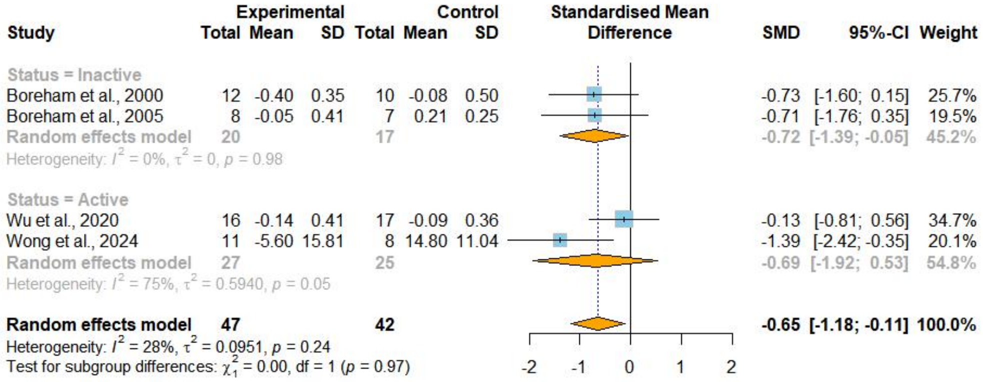

Figure S6 Meta-analysis of ExSn versus controls on total cholesterol, using SMD to indicate the difference in change values between ExSn and control groups. Subgroup analysis based on participants' physical activity level (active vs. inactive).

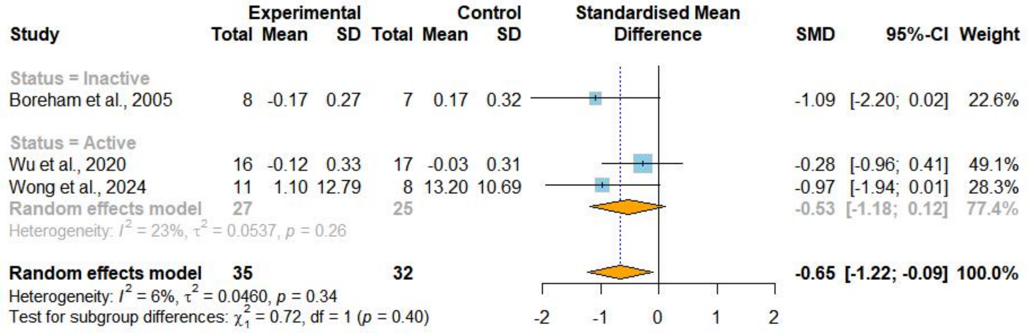

Figure S7 Meta-analysis of ExSn versus controls on low-density lipoprotein cholesterol, using SMD to indicate the difference in change values between ExSn and control groups. Subgroup analysis based on participants' physical activity level (active vs. inactive).

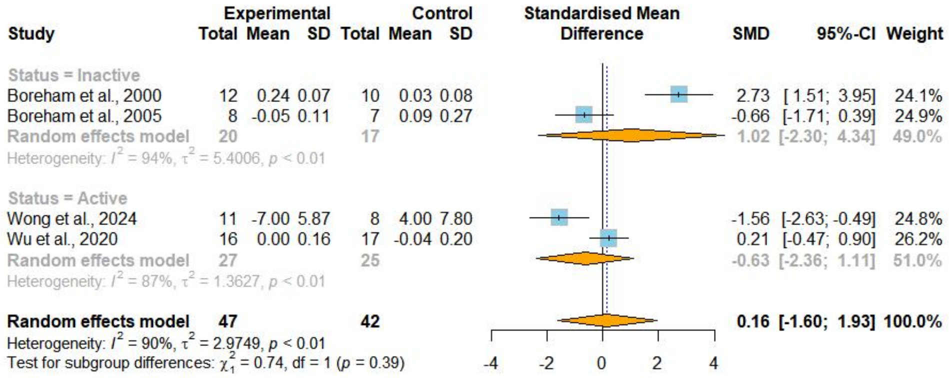

Figure S8 Meta-analysis of ExSn versus controls on high-density lipoprotein cholesterol, using SMD to indicate the difference in change values between ExSn and control groups. Subgroup analysis based on participants' physical activity level (active vs. inactive).

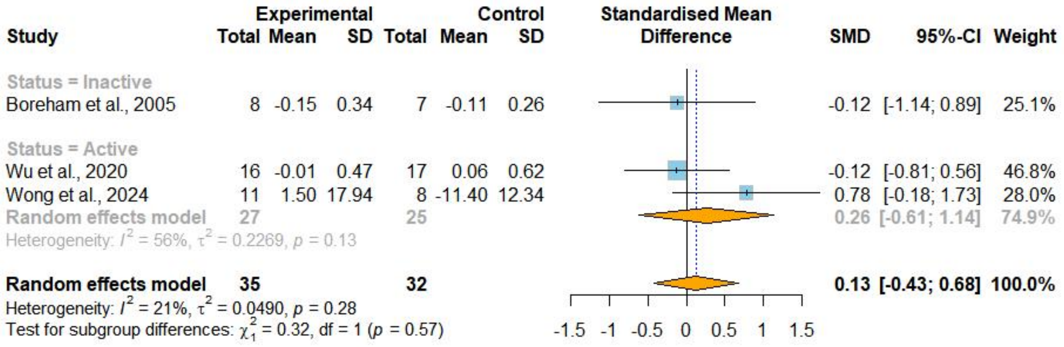

Figure S9 Meta-analysis of ExSn versus controls on triglycerides, using SMD to indicate the difference in change values between ExSn and control groups. Subgroup analysis based on participants' physical activity level (active vs. inactive).

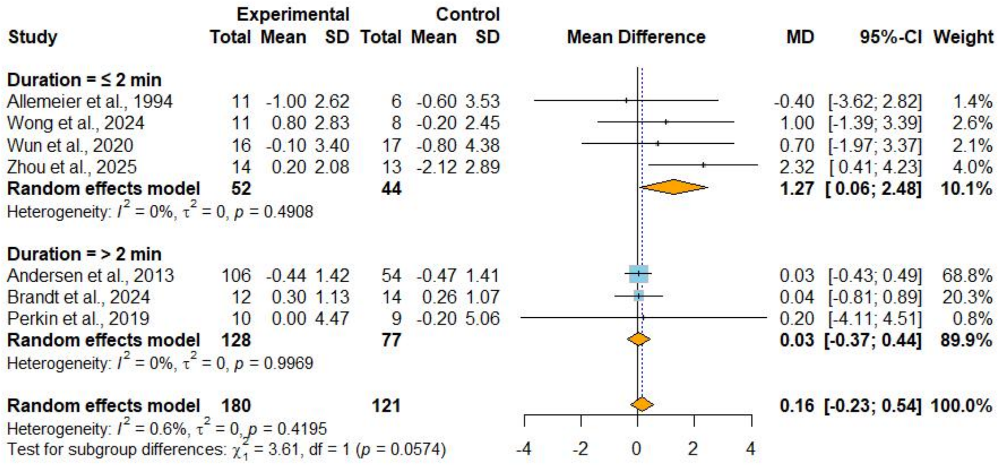

Figure S10 Meta-analysis of ExSn versus controls on body fat, using MD to indicate the difference in change values between ExSn and control groups. Subgroup analysis based on the duration for each bout of ExSn (≤ 2 min vs. > 2 min).

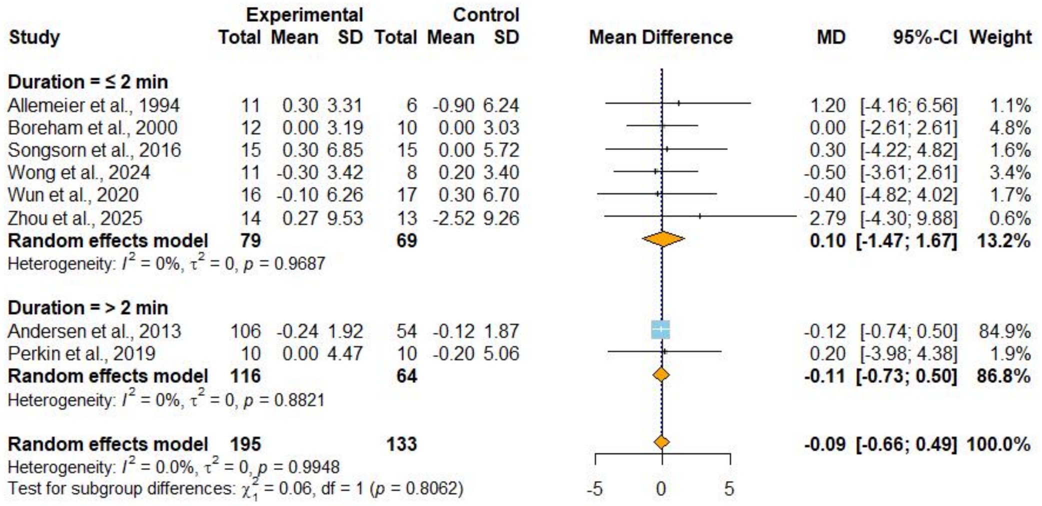

Figure S11 Meta-analysis of ExSn versus controls on body weight, using MD to indicate the difference in change values between ExSn and control groups. Subgroup analysis based on the duration for each bout of ExSn (≤ 2 min vs. > 2 min).

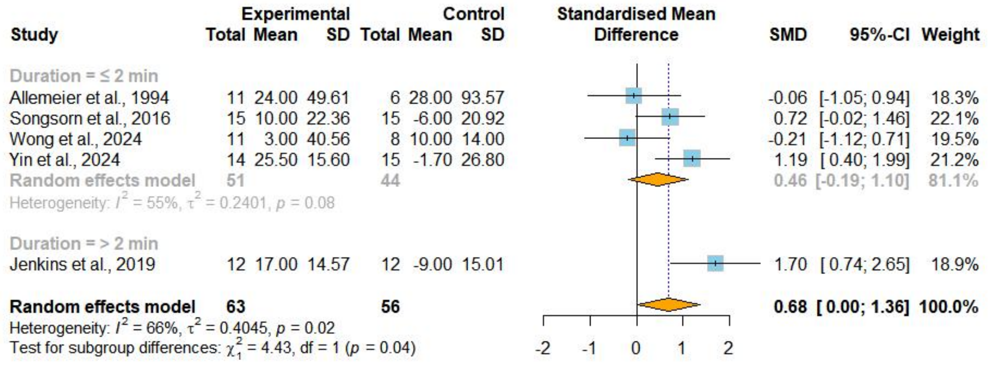

Figure S12 Meta-analysis of ExSn versus controls on peak power output, using SMD to indicate the difference in change values between ExSn and control groups. Subgroup analysis based on the duration for each bout of ExSn (≤ 2 min vs. > 2 min).

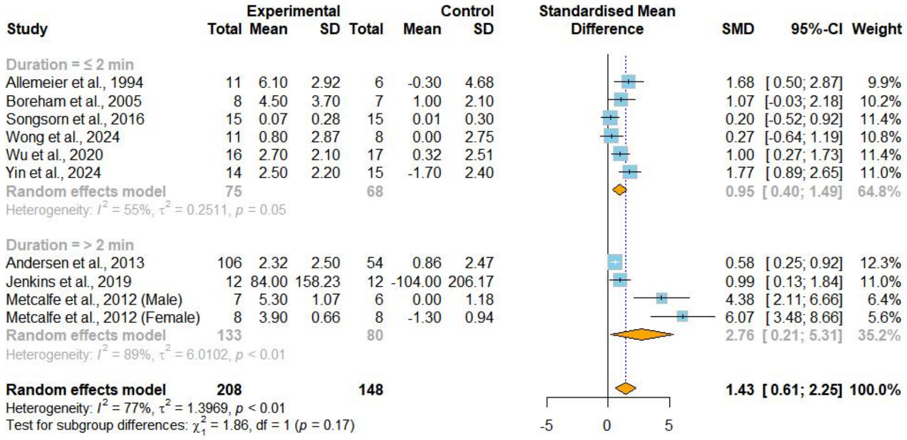

Figure S13 Meta-analysis of ExSn versus controls on maximal oxygen uptake, using SMD to indicate the difference in change values between ExSn and control groups. Subgroup analysis based on the duration for each bout of ExSn (≤ 2 min vs. > 2 min).

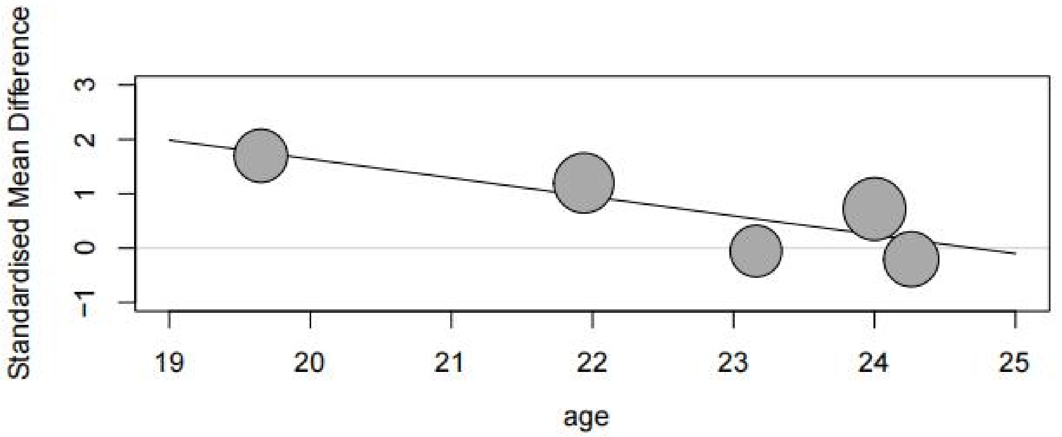

Figure S14 Meta-regression of peak power output moderated by age

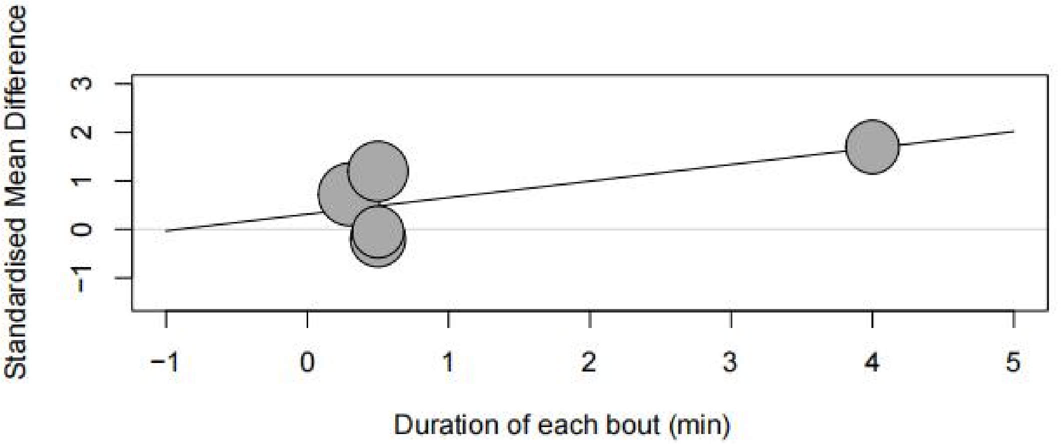

Figure S15 Meta-regression of peak power output moderated by the duration of each ExSn bout

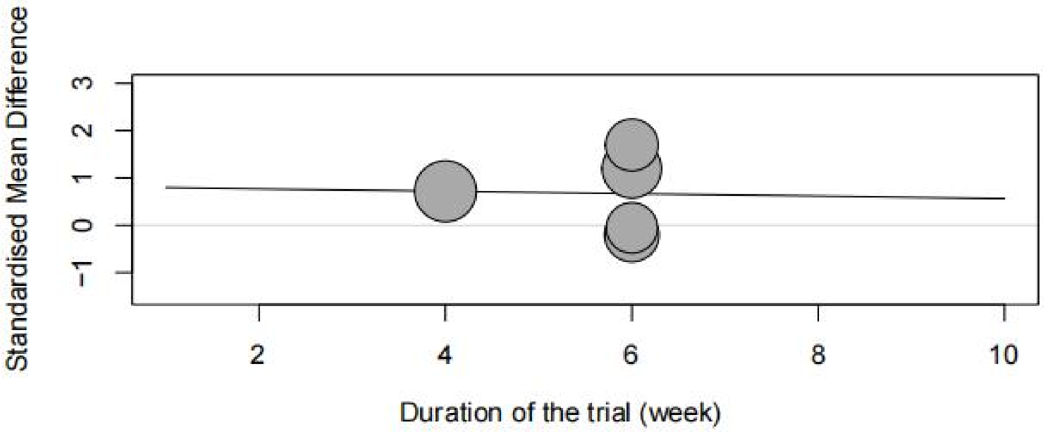

Figure S16 Meta-regression of peak power output moderated by the duration of the trial

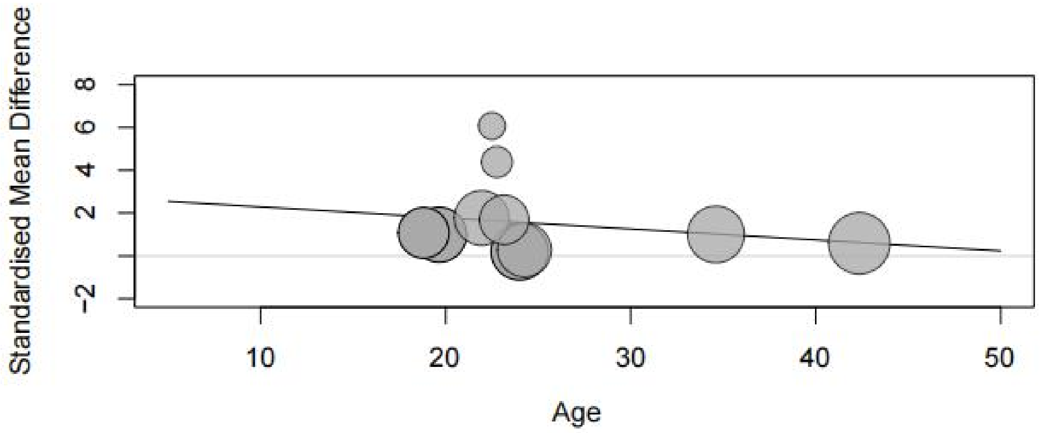

Figure S17 Meta-regression of maximal oxygen uptake moderated by age

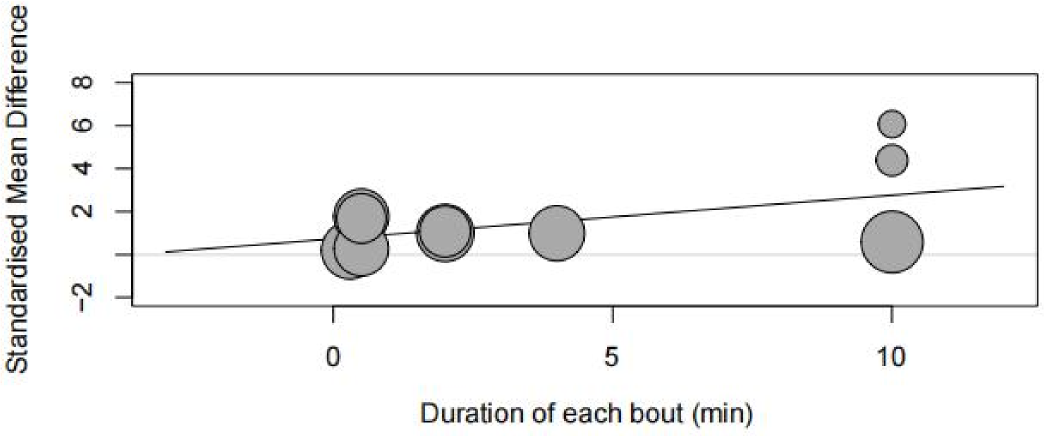

Figure S18 Meta-regression of maximal oxygen uptake moderated by the duration of each ExSn bout

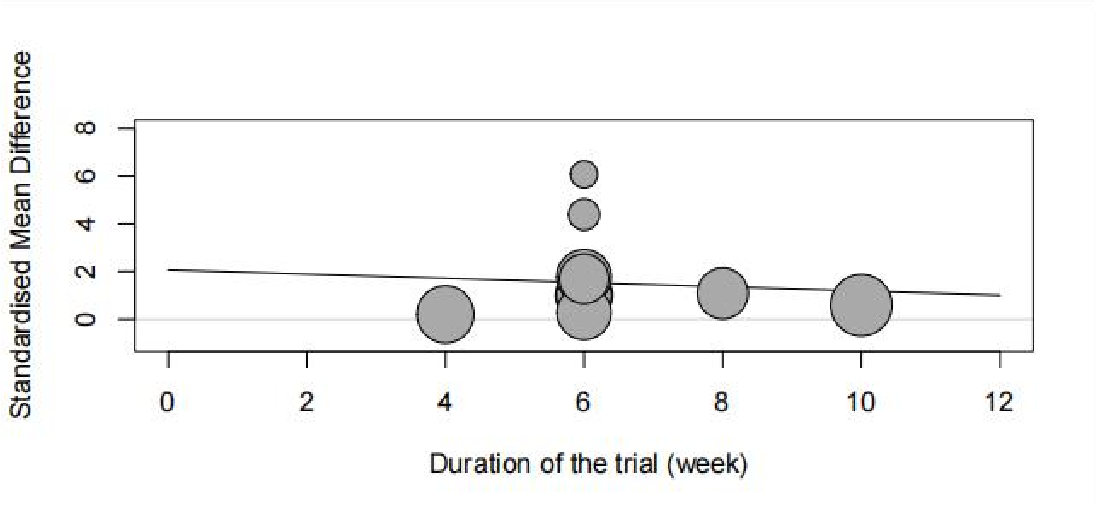

Figure S19 Meta-regression of maximal oxygen uptake moderated by the duration of the trial
